# Supplementary material for: Brown adipose tissue activity impacts systemic lactate clearance in male mice
Source: J Physiol. 2025 Sep 22;603(22):6811–32. doi: 10.1113/JP288871 (PMC12645562; doi:10.1113/JP288871)
Supplement: Supplementary file 2 — Supporting Information [file TJP-603-6811-s001.pdf]

Table S1A. Plasma

			m+0		m+1		m+2		m+3		m+4		m+5		m+6	
	Condition	mean enrichment	Corrected area	fraction	Corrected area	fraction	Corrected area	fraction	Corrected area	fraction	Corrected area	fraction	Corrected area	fraction	Corrected area	fraction
lactate	4°C	0,14572504	1,03E+09	0,83745	2,05E+07	0,01670533	2,10E+07	0,01707089	1,58E+08	0,128776						
		0,05320301	1,92E+09	0,93674	1,92E+07	0,00933726	2,36E+07	0,01149016	8,71E+07	0,04243049						
		0,14834725	1,49E+09	0,83522	2,87E+07	0,01611529	3,04E+07	0,01705933	2,35E+08	0,1316026						
		0,11773255	2,12E+09	0,8673	3,66E+07	0,01493557	3,68E+07	0,01504119	2,51E+08	0,10272656						
		0,16397534	1,42E+09	0,81898	2,81E+07	0,01619267	3,25E+07	0,01873747	2,53E+08	0,14608614						
	21°C	0,12220939	2,11E+09	0,86389	3,32E+07	0,01359342	3,55E+07	0,01451916	2,64E+08	0,10799881						
		0,11313964	2,47E+09	0,87353	3,78E+07	0,01337755	3,74E+07	0,01324104	2,82E+08	0,09985309						
		0,12832357	1,54E+09	0,85354	3,20E+07	0,01771327	3,42E+07	0,01897145	1,98E+08	0,10977152						
		0,08692922	2,42E+09	0,90069	3,67E+07	0,01364274	2,65E+07	0,00986091	2,04E+08	0,0758077						
		0,13531582	1,83E+09	0,84688	3,63E+07	0,01684637	4,25E+07	0,01971081	2,51E+08	0,11655982						
	30°C	0,16409628	6,52E+08	0,81217	1,82E+07	0,02270446	2,07E+07	0,02578861	1,12E+08	0,13933572						
		0,26239819	1,63E+09	0,71881	4,28E+07	0,01886906	4,22E+07	0,0186287	5,53E+08	0,24368937						
		0,08183796	2,49E+09	0,90361	3,93E+07	0,01422156	4,20E+07	0,01521309	1,85E+08	0,06695538						
		0,19023368	1,76E+09	0,79358	3,38E+07	0,0151935	4,04E+07	0,0181854	3,85E+08	0,17304558						
		0,169372	1,34E+09	0,81163	3,10E+07	0,01882961	3,18E+07	0,01933351	2,47E+08	0,15020645						
pyruvate	4°C	0,132299602	7,62E+07	0,853562865	1,23E+06	0,013829791	1,32E+06	0,014753017	1,05E+07	0,117854327						
		0,047215659	1,36E+08	0,94620302	6,06E+05	0,00420196	1,64E+06	0,011340043	5,52E+06	0,038254977						
		0,148256888	9,97E+07	0,833791977	2,15E+06	0,017951068	2,15E+06	0,017951268	1,56E+07	0,130305687						
		0,114532458	1,31E+08	0,869090821	2,59E+06	0,017211294	2,21E+06	0,014707573	1,49E+07	0,098990311						
		0,154573077	1,11E+08	0,825655735	2,69E+06	0,020045432	2,58E+06	0,019222701	1,81E+07	0,135076133						
	21°C	0,120126774	1,32E+08	0,863520761	2,77E+06	0,018189449	1,93E+06	0,012678493	1,61E+07	0,105611296						
		0,108968617	1,17E+08	0,877797921	1,98E+06	0,014824372	1,34E+06	0,010051641	1,30E+07	0,097326065						
		0,117129806	8,91E+07	0,86966831	1,10E+06	0,010779686	1,85E+06	0,018046279	1,04E+07	0,101505724						
		0,081739961	1,54E+08	0,906076862	2,37E+06	0,013902015	1,49E+06	0,0087455	1,21E+07	0,071275623						
		0,130544914	1,05E+08	0,852064806	1,92E+06	0,015654267	2,56E+06	0,020862304	1,37E+07	0,111418622						
	30°C	0,158800685	5,28E+07	0,819683494	1,36E+06	0,021171138	1,43E+06	0,022205189	8,82E+06	0,13694018						
		0,241351384	1,01E+08	0,743222701	1,79E+06	0,013233748	2,69E+06	0,019810249	3,03E+07	0,223733302						
		0,076041827	1,68E+08	0,909259848	2,61E+06	0,01412417	2,92E+06	0,015846634	1,12E+07	0,060769348						
		0,178604073	1,01E+08	0,808245975	1,41E+06	0,011301011	2,11E+06	0,016847834	2,04E+07	0,163605181						
		0,165614795	1,01E+08	0,814195554	2,52E+06	0,020300749	2,48E+06	0,019967456	1,80E+07	0,145536241						
glucose	4°C	0,071712861	1,72E+08	0,818154203	9,87E+06	0,046844287	1,13E+07	0,053698285	1,29E+07	0,061231313	2,15E+06	0,01021364	1,61E+06	0,007661821	4,63E+05	0,00219645
		0,051784607	2,44E+08	0,862221992	1,11E+07	0,03931785	1,10E+07	0,039031851	1,41E+07	0,049927848	1,39E+06	0,004917746	1,03E+06	0,003624717	2,71E+05	0,000957996
		0,100752275	1,58E+08	0,742192041	1,42E+07	0,066718556	1,71E+07	0,080603571	1,75E+07	0,082275115	3,17E+06	0,014929433	2,05E+06	0,009642826	7,73E+05	0,003638457
		0,057224479	2,95E+08	0,847090624	1,65E+07	0,047479261	1,45E+07	0,04173307	1,75E+07	0,050264445	2,41E+06	0,006926913	1,79E+06	0,00513364	4,78E+05	0,001372048
		0,102431641	1,69E+08	0,73688716	1,53E+07	0,066494367	1,96E+07	0,085257271	1,96E+07	0,085216507	2,82E+06	0,012261719	2,40E+06	0,01041331	7,98E+05	0,003469665
	21°C	0,029807457	2,28E+08	0,920738233	5,83E+06	0,023515053	4,36E+06	0,017571072	8,39E+06	0,033824196	7,96E+05	0,00321115	2,41E+05	0,000971421	4,19E+04	0,000168875
		0,047113083	2,00E+08	0,87574854	7,45E+06	0,032681124	8,23E+06	0,036136569	1,08E+07	0,047218804	1,19E+06	0,005213493	6,37E+05	0,002794962	4,71E+04	0,000206507
		0,051525535	2,85E+08	0,86416006	1,24E+07	0,037618787	1,35E+07	0,040865194	1,52E+07	0,046277467	1,92E+06	0,005817227	1,27E+06	0,00386486	4,60E+05	0,001396405
		0,018572208	2,93E+08	0,94453287	6,85E+06	0,022085281	3,57E+06	0,0114965	6,57E+06	0,021186431	2,17E+05	0,000698919	0,00E+00	0	0,00E+00	0
		0,067121653	2,82E+08	0,826084896	1,63E+07	0,047823817	1,74E+07	0,05093722	2,01E+07	0,058785111	2,59E+06	0,007579696	2,18E+06	0,006378018	8,23E+05	0,002411243
	30°C	0,057333432	9,22E+07	0,845053592	4,66E+06	0,042722964	5,27E+06	0,048268262	6,01E+06	0,055109032	5,80E+05	0,005314567	3,31E+05	0,003033758	5,43E+04	0,000497826
		0,079355721	2,18E+08	0,802751217	1,22E+07	0,044840884	1,62E+07	0,05964023	1,96E+07	0,072363088	2,70E+06	0,009972609	2,05E+06	0,007558547	7,79E+05	0,002873424
		0,041774018	2,69E+08	0,883140385	1,14E+07	0,037269028	1,06E+07	0,034614964	1,16E+07	0,037997941	1,46E+06	0,004783686	6,54E+05	0,002147391	1,42E+04	4,66E-05
		0,032901331	2,04E+08	0,912179919	5,31E+06	0,023706606	5,63E+06	0,025139693	7,53E+06	0,033626455	9,98E+05	0,00445572	1,41E+05	0,000629891	5,86E+04	0,000261717
		0,046607281	1,96E+08	0,87790573	7,39E+06	0,033123409	7,83E+06	0,035074104	9,80E+06	0,043919142	1,33E+06	0,005942018	7,51E+05	0,003367011	1,49E+05	0,000668585

Table S1B. BAT

			m+0		m+1		m+2		m+3		m+4		m+5		m+6	
	Condition	mean enrichment	Corrected area	fraction	Corrected area	fraction	Corrected area	fraction	Corrected area	fraction	Corrected area	fraction	Corrected area	fraction	Corrected area	fraction
lactate	4°C	0,08227937	1,16E+09	0,88372922	4,65E+07	0,03543957	4,08E+07	0,03109508	6,53E+07	0,04973613						
		0,05414475	2,44E+09	0,91960937	6,81E+07	0,02563961	7,30E+07	0,02745843	7,25E+07	0,02729259						
		0,09907004	1,79E+09	0,85800152	9,13E+07	0,04363138	8,69E+07	0,04152256	1,19E+08	0,05684454						
		0,07897146	2,86E+09	0,87856748	1,48E+08	0,0454704	1,19E+08	0,03644236	1,29E+08	0,03951975						
		0,10080307	2,31E+09	0,84775447	1,53E+08	0,05610589	1,15E+08	0,0421156	1,47E+08	0,05402405						
	21°C	0,05758592	1,19E+09	0,91872781	3,35E+07	0,02591127	2,48E+07	0,01923627	4,67E+07	0,03612465						
		0,07021967	2,05E+09	0,89616992	8,54E+07	0,03735182	5,97E+07	0,02612759	9,23E+07	0,04035067						
		0,07040917	2,24E+09	0,89369026	9,33E+07	0,03726518	8,31E+07	0,03317136	8,98E+07	0,0358732						
		0,04479802	1,36E+09	0,93913504	2,49E+07	0,01713725	2,02E+07	0,01392631	4,32E+07	0,0298014						
		0,0855311	1,47E+09	0,88023325	5,82E+07	0,03472896	5,57E+07	0,03324904	8,67E+07	0,05178876						
	30°C	0,12201061	1,40E+09	0,84406427	5,74E+07	0,03461964	5,40E+07	0,03253608	1,47E+08	0,08878001						
		0,17086363	7,36E+08	0,79997306	2,65E+07	0,02884496	2,74E+07	0,02980001	1,30E+08	0,14138197						
		0,05328776	9,98E+08	0,93105797	1,69E+07	0,0157626	1,65E+07	0,0154376	4,05E+07	0,03774182						
		0,08823436	8,76E+08	0,88409093	2,99E+07	0,0301918	2,24E+07	0,02264052	6,25E+07	0,06307675						
		0,1052601	9,43E+08	0,86915587	2,77E+07	0,02556193	2,78E+07	0,02562825	8,64E+07	0,07965396						
pyruvate	4°C	0,06238293	9,56E+06	0,91976	1,99E+05	0,01916	1,59E+05	0,01525587	4,76E+05	0,04582592						
		0,04554486	2,30E+07	0,9351	4,76E+05	0,01936	4,75E+05	0,01934321	6,44E+05	0,026195						
		0,08735199	1,55E+07	0,8788	6,30E+05	0,03568	5,32E+05	0,03017046	9,77E+05	0,05534525						
		0,06403334	2,30E+07	0,90367	9,01E+05	0,03535	6,67E+05	0,02619834	8,86E+05	0,03478346						
		0,10131163	2,24E+07	0,85814	1,14E+06	0,04368	8,95E+05	0,03427638	1,67E+06	0,06389962						
	21°C	0,0533168	1,86E+07	0,93278	2,82E+05	0,0141	2,70E+05	0,01351395	7,91E+05	0,03960612						
		0,03412343	1,14E+07	0,95231	1,67E+05	0,01387	1,56E+05	0,01296416	2,51E+05	0,02085662						
		0,05819176	2,28E+07	0,91886	5,56E+05	0,02245	5,93E+05	0,02396295	8,60E+05	0,03473478						
		0,04196415	1,56E+07	0,94702	2,14E+05	0,01299	1,16E+05	0,00705526	5,41E+05	0,03293022						
		0,06998068	1,97E+07	0,90537	5,65E+05	0,026	4,77E+05	0,02195128	1,01E+06	0,04667965						
	30°C	0,12113797	6,73E+07	0,84591	2,68E+06	0,03371	2,50E+06	0,03142471	7,07E+06	0,088951						
		0,14700753	1,69E+07	0,82821	4,79E+05	0,02352	5,55E+05	0,02729011	2,46E+06	0,12097248						
		0,03765631	7,85E+06	0,95657	3,92E+04	0,00477	6,39E+04	0,00778432	2,54E+05	0,03087536						
		0,04526884	5,73E+06	0,95082	3,26E+04	0,00541	5,45E+03	0,00090383	2,58E+05	0,04286344						
		0,10801711	1,84E+07	0,87148	4,41E+05	0,02094	4,13E+05	0,01961458	1,85E+06	0,0879606						
CisAco	4°C	0,0246668	1,93E+07	0,89814	1,47E+06	0,06817984	4,56E+05	0,02120725	2,68E+05	0,01246881	0,00E+00	0	0,00E+00	0	0,00E+00	0
		0,0096321	1,24E+07	0,96271	2,99E+05	0,0231578	1,18E+05	0,00911205	5,80E+04	0,00449204	1,65E+03	0,00012803	0,00E+00	0	5,21E+03	0,00040374
		0,01743005	1,17E+07	0,93064	5,49E+05	0,04378453	2,00E+05	0,01592451	1,21E+05	0,00964893	0,00E+00	0	0,00E+00	0	0,00E+00	0
		0,0245127	1,63E+07	0,89421	1,30E+06	0,07141334	5,06E+05	0,02783914	1,15E+05	0,00634412	0,00E+00	0	3,46E+03	0,00019045	0,00E+00	0
		0,02318357	1,02E+07	0,91439	5,16E+05	0,04630347	2,80E+05	0,02510975	1,58E+05	0,01419281	0,00E+00	0	0,00E+00	0	0,00E+00	0
	21°C	0,01116006	9,48E+06	0,95444	2,78E+05	0,02794559	1,44E+05	0,01452608	2,36E+04	0,00237819	7,02E+03	0,00070702	0,00E+00	0	0,00E+00	0
		0,00579314	8,51E+06	0,9803	1,31E+05	0,01511305	0,00E+00	0	1,87E+04	0,00215412	6,11E+03	0,00070422	0,00E+00	0	1,50E+04	0,00172776
		0,01391633	8,29E+06	0,93931	3,36E+05	0,03802608	1,99E+05	0,02252544	1,24E+03	0,00014033	0,00E+00	0	0,00E+00	0	0,00E+00	0
		0,01272416	7,17E+06	0,94171	3,43E+05	0,04505826	8,04E+04	0,01055802	1,49E+04	0,00196264	0,00E+00	0	0,00E+00	0	5,44E+03	0,00071379
		0,01967271	1,37E+07	0,92166	7,42E+05	0,04984573	2,63E+05	0,0176887	1,58E+05	0,0105963	0,00E+00	0	3,05E+03	0,00020484	0,00E+00	0
	30°C	0,03912825	9,03E+06	0,86174	6,74E+05	0,06428529	5,69E+05	0,05432184	1,87E+05	0,01786582	1,19E+04	0,00113586	2,01E+03	0,00019139	4,79E+03	0,00045711
		0,02190233	8,52E+06	0,92783	3,07E+05	0,03346993	2,05E+05	0,02229979	1,23E+05	0,01340221	2,24E+04	0,00243316	0,00E+00	0	5,21E+03	0,00056754
		0,0018567	8,39E+06	0,98886	9,45E+04	0,0111402	0,00E+00	0	0,00E+00	0	0,00E+00	0	0,00E+00	0	0,00E+00	0
		0,00194907	8,19E+06	0,99352	4,19E+04	0,00508779	0,00E+00	0	1,24E+03	0,0001506	5,41E+03	0,00065606	0,00E+00	0	4,85E+03	0,00058843
		0,01734559	7,24E+06	0,93201	3,04E+05	0,03921948	1,67E+05	0,0214697	5,67E+04	0,00730488	0,00E+00	0	0,00E+00	0	0,00E+00	0
Akg	4°C	0,02618	1,25E+07	0,91822632	5,12E+05	0,03757546	5,35E+05	0,03928789	6,69E+04	0,00491033	0,00E+00	0	0,00E+00	0		
		0,01503	1,92E+07	0,951189	4,87E+05	0,02413623	4,71E+05	0,02332683	2,06E+04	0,00102251	6,57E+03	0,00032543	0,00E+00	0		
		0,04237	2,06E+07	0,87482925	1,31E+06	0,05535321	1,27E+06	0,05363037	3,66E+05	0,01552473	1,56E+04	0,00066243	0,00E+00	0		
		0,03041	1,12E+07	0,89644667	7,33E+05	0,0587201	5,23E+05	0,04190844	3,19E+04	0,00255344	0,00E+00	0	4,63E+03	0,00037135		
		0,0558	2,34E+07	0,84318191	1,59E+06	0,05749305	2,14E+06	0,07726692	6,01E+05	0,02164664	0,00E+00	0	1,14E+04	0,00041148		
	21°C	0,02275	1,44E+07	0,93541039	3,58E+05	0,02323177	5,64E+05	0,03660183	4,30E+04	0,00278976	1,35E+04	0,00087448	1,68E+04	0,00109178		
		0,00711	8,06E+06	0,97536916	1,13E+05	0,01369306	9,04E+04	0,01093778	0,00E+00	0	0,00E+00	0	0,00E+00	0		
		0,04135	1,68E+07	0,87217331	1,17E+06	0,06073258	1,09E+06	0,05631219	1,88E+05	0,00974103	2,01E+04	0,00104089	0,00E+00	0		
		0,05461	2,94E+07	0,8475193	1,84E+06	0,05311343	2,86E+06	0,08251514	4,51E+05	0,01300338	1,17E+05	0,00336276	1,69E+04	0,000486		
		0,04133	2,64E+07	0,8783389	1,55E+06	0,05149096	1,66E+06	0,05534964	4,45E+05	0,0148205	0,00E+00	0	0,00E+00	0		
	30°C	0,07214	2,07E+07	0,80995268	1,52E+06	0,05937947	2,51E+06	0,09819502	6,74E+05	0,02635476	1,21E+05	0,00474358	3,51E+04	0,00137449		
		0,06931	3,20E+07	0,8163606	2,35E+06	0,06003079	3,60E+06	0,09188212	9,84E+05	0,02509611	2,23E+05	0,00567563	3,74E+04	0,00095475		

		0,02364	1,62E+07	0,92919268	4,89E+05	0,0279722	6,69E+05	0,03826434	7,99E+04	0,00457078	0,00E+00	0	0,00E+00	0
		0,02906	7,95E+06	0,92327754	1,92E+05	0,02226922	3,68E+05	0,04267886	8,13E+04	0,00944156	2,01E+04	0,00233281	0,00E+00	0
		0,07294	2,01E+07	0,80908739	1,42E+06	0,05731198	2,52E+06	0,10128731	6,50E+05	0,02615465	1,11E+05	0,00446873	4,20E+04	0,00168994
Gln	4°C	0,05165801	5,15E+08	0,84938008	4,07E+07	0,06718357	3,81E+07	0,06281379	1,07E+07	0,01764201	1,42E+06	0,00234988	3,82E+05	0,00063067
		0,03654791	6,49E+08	0,89031752	3,60E+07	0,04941002	3,51E+07	0,04811343	8,42E+06	0,01154549	4,39E+05	0,00060151	8,78E+03	1,20E-05
		0,07240251	4,38E+08	0,79699119	4,54E+07	0,08276155	4,75E+07	0,08653616	1,62E+07	0,02955531	1,79E+06	0,0032662	4,88E+05	0,00088959
		0,06588034	5,85E+08	0,80215868	6,90E+07	0,0946363	5,81E+07	0,07972415	1,41E+07	0,01935525	2,46E+06	0,0033768	5,46E+05	0,00074883
		0,07447662	5,27E+08	0,79641162	5,18E+07	0,07830894	5,85E+07	0,08841493	2,08E+07	0,03151283	2,68E+06	0,00405263	8,59E+05	0,00129905
	21°C	0,04413169	1,16E+08	0,87788755	6,18E+06	0,04696208	7,46E+06	0,05670176	1,88E+06	0,01426142	4,51E+05	0,00342736	1,00E+05	0,00075982
		0,05167297	3,76E+08	0,85648979	2,53E+07	0,05765591	2,77E+07	0,06311079	7,77E+06	0,01769173	1,69E+06	0,00384673	5,29E+05	0,00120505
		0,06041117	4,58E+08	0,8271719	4,02E+07	0,07275115	4,17E+07	0,07530868	1,17E+07	0,02119496	1,53E+06	0,00276408	4,48E+05	0,00080923
		0,05489717	3,06E+08	0,84657943	2,23E+07	0,06180732	2,49E+07	0,0690792	6,13E+06	0,01699019	1,50E+06	0,00416973	4,96E+05	0,00137413
		0,05177931	5,53E+08	0,85257988	3,99E+07	0,06144058	4,15E+07	0,06390808	1,25E+07	0,01929015	1,39E+06	0,00213723	4,18E+05	0,00064408
	30°C	0,06466603	1,77E+08	0,83755653	1,02E+07	0,04820739	1,69E+07	0,07978843	5,24E+06	0,02475264	1,52E+06	0,0071871	5,31E+05	0,00250792
		0,089839	1,65E+08	0,77917759	1,30E+07	0,06135347	2,32E+07	0,1094309	7,44E+06	0,03507688	2,34E+06	0,0110567	8,28E+05	0,00390447
		0,03891184	1,83E+08	0,8959451	6,99E+06	0,03421016	1,07E+07	0,05235825	3,04E+06	0,01488907	4,13E+05	0,00202179	1,18E+05	0,00057564
		0,05126385	5,71E+07	0,86669261	2,85E+06	0,04323196	4,29E+06	0,06516031	1,23E+06	0,01862188	3,01E+05	0,00456513	1,14E+05	0,0017281
		0,05516077	1,05E+08	0,85779368	5,23E+06	0,04265268	8,86E+06	0,07216768	2,68E+06	0,02186337	5,38E+05	0,00438721	1,39E+05	0,00113537
Glu	4°C	0,03831613	3,12E+08	0,87951864	2,20E+07	0,06194825	1,68E+07	0,0473005	3,53E+06	0,00996802	4,24E+05	0,00119562	2,45E+04	6,90E-05
		0,02185718	2,25E+08	0,93025346	8,47E+06	0,03507487	7,21E+06	0,02988203	1,14E+06	0,0047116	1,88E+04	7,80E-05	0,00E+00	0
		0,05518693	2,46E+08	0,83543842	2,21E+07	0,07478041	2,07E+07	0,07015783	5,26E+06	0,01782597	4,80E+05	0,00162619	5,05E+04	0,00017118
		0,05312918	1,89E+08	0,83294836	1,93E+07	0,08491226	1,53E+07	0,06751551	2,92E+06	0,01285951	3,86E+05	0,00169768	1,52E+04	6,67E-05
		0,04623153	1,94E+08	0,8650229	1,28E+07	0,05683737	1,38E+07	0,06123503	3,57E+06	0,01590802	1,93E+05	0,00085723	3,13E+04	0,00013945
	21°C	0,0267023	5,13E+08	0,9242339	1,69E+07	0,03053877	1,95E+07	0,03521183	4,36E+06	0,007864	9,99E+05	0,00180042	1,95E+05	0,00035108
		0,03089614	3,95E+08	0,91105292	1,64E+07	0,03783914	1,70E+07	0,03926558	4,20E+06	0,00967414	7,60E+05	0,00175307	1,80E+05	0,00041514
		0,03581749	2,22E+08	0,89150595	1,20E+07	0,04840088	1,26E+07	0,05063521	2,10E+06	0,00843425	2,50E+05	0,00100517	4,61E+03	1,85E-05
		0,03313301	1,84E+08	0,90228284	8,22E+06	0,04021173	9,97E+06	0,04876117	1,46E+06	0,00713636	3,10E+05	0,00151759	1,85E+04	9,03E-05
		0,03635215	3,26E+08	0,88867804	1,94E+07	0,05288852	1,73E+07	0,04729254	3,77E+06	0,01027648	3,17E+05	0,00086442	0,00E+00	0
	30°C	0,03233415	1,65E+08	0,91010623	5,28E+06	0,02906184	9,40E+06	0,05171977	1,33E+06	0,0073023	3,25E+05	0,00178686	4,18E+03	2,30E-05
		0,03898643	1,09E+08	0,89442871	4,20E+06	0,03449469	6,82E+06	0,05607914	1,45E+06	0,01194342	3,43E+05	0,00282129	2,83E+04	0,00023276
		0,01795856	2,85E+08	0,9467766	6,44E+06	0,02137924	8,22E+06	0,02730948	1,31E+06	0,00434414	5,74E+04	0,00019054	0,00E+00	0
		0,03464215	2,57E+08	0,90675964	9,15E+06	0,03222662	1,32E+07	0,04665946	3,03E+06	0,01067056	7,85E+05	0,00276507	2,61E+05	0,00091864
		0,02882498	1,96E+08	0,92207299	5,14E+06	0,02416929	9,23E+06	0,04336181	1,84E+06	0,00864318	3,11E+05	0,00146122	6,20E+04	0,00029151
Succ	4°C	0,05696381	1,48E+09	0,85007364	1,57E+08	0,09007695	7,52E+07	0,04326151	2,62E+07	0,01509634	2,59E+06	0,00149156		
		0,04469273	1,55E+09	0,88099815	1,24E+08	0,07043056	6,66E+07	0,03787268	1,79E+07	0,01019943	8,77E+05	0,00049917		
		0,09038864	1,01E+09	0,76828729	1,72E+08	0,13042555	1,00E+08	0,0758451	2,94E+07	0,02232942	4,10E+06	0,00311264		
		0,07898715	1,42E+09	0,78765027	2,33E+08	0,12911251	1,17E+08	0,06473032	3,00E+07	0,01665214	3,34E+06	0,00185476		
		0,08878291	1,10E+09	0,77453181	1,81E+08	0,12713147	9,97E+07	0,07011064	3,57E+07	0,02512545	4,41E+06	0,00310063		
	21°C	0,05723471	1,72E+09	0,84683476	1,89E+08	0,09336116	9,24E+07	0,04556467	2,54E+07	0,0125093	3,51E+06	0,00173011		
		0,06746721	1,36E+09	0,82256859	1,75E+08	0,10525081	9,01E+07	0,05428059	2,58E+07	0,01554318	3,91E+06	0,00235682		
		0,06593378	1,66E+09	0,82408395	2,14E+08	0,10615677	1,08E+08	0,05331693	2,99E+07	0,01482494	3,26E+06	0,00161742		
		0,04680779	9,65E+08	0,87300646	8,76E+07	0,07921243	4,06E+07	0,03673417	1,07E+07	0,00963736	1,56E+06	0,00140958		
		0,05790521	1,35E+09	0,8481312	1,40E+08	0,08845085	7,70E+07	0,04848914	2,15E+07	0,01352353	2,23E+06	0,00140528		
	30°C	0,09031995	7,51E+08	0,77030128	1,23E+08	0,12606295	7,74E+07	0,07934253	2,01E+07	0,02064118	3,56E+06	0,00365207		
		0,05970843	3,99E+08	0,8564693	3,28E+07	0,07034664	2,49E+07	0,05346301	8,07E+06	0,01732314	1,12E+06	0,00239792		
		0,03028479	1,00E+09	0,91955102	5,16E+07	0,04742721	2,78E+07	0,0255162	7,99E+06	0,00734271	1,77E+05	0,00016286		
		0,07095287	7,06E+08	0,81925799	8,61E+07	0,09982547	5,31E+07	0,06163165	1,42E+07	0,01641686	2,47E+06	0,00286803		
		0,05252104	9,06E+08	0,86295709	8,29E+07	0,07894146	4,67E+07	0,04450528	1,29E+07	0,01225252	1,41E+06	0,00134365		
Asp	4°C	0,03631917	1,99E+08	0,90315378	1,31E+07	0,05933257	5,96E+06	0,02709423	2,18E+06	0,00992204	1,09E+05	0,00049738		
		0,02769921	2,64E+08	0,92145188	1,54E+07	0,05380937	4,94E+06	0,01722875	2,15E+06	0,00751	0,00E+00	0		
		0,06276194	1,70E+08	0,83870385	1,89E+07	0,09315803	9,81E+06	0,04837829	3,63E+06	0,01790619	3,76E+05	0,00185364		
		0,05227822	2,01E+08	0,85415602	2,22E+07	0,0944195	9,46E+06	0,04018677	2,50E+06	0,01063098	1,43E+05	0,00060673		
		0,05869936	1,94E+08	0,84048587	2,42E+07	0,10475048	8,15E+06	0,03533192	4,23E+06	0,01834377	2,51E+05	0,00108795		
	21°C	0,01126494	3,87E+08	0,96962108	7,72E+06	0,01937401	2,95E+06	0,00739009	1,42E+06	0,00355371	2,44E+04	6,11E-05		
		0,0192844	4,34E+08	0,94844426	1,49E+07	0,03258136	5,80E+06	0,01266977	2,75E+06	0,00600176	1,39E+05	0,00030285		
		0,04425353	2,49E+08	0,88177332	2,04E+07	0,07226382	9,49E+06	0,03362995	3,34E+06	0,01184122	1,39E+05	0,00049168		
		0,02496158	1,21E+08	0,93112687	5,60E+06	0,04316454	2,66E+06	0,02049058	6,71E+05	0,00517144	6,04E+03	4,66E-05		
		0,03139325	1,88E+08	0,91659157	1,02E+07	0,04997653	5,15E+06	0,02517043	1,59E+06	0,00779027	9,64E+04	0,00047119		
	30°C	0,02619419	1,19E+08	0,93425669	4,40E+06	0,0345039	3,05E+06	0,02386394	8,88E+05	0,00695692	5,34E+04	0,00041855		
		0,02409058	6,32E+07	0,94477264	1,58E+06	0,02361595	1,48E+06	0,02209899	6,36E+05	0,00950126	7,47E+02	1,12E-05		
		0,01664874	2,03E+08	0,96026495	4,09E+06	0,01933523	2,95E+06	0,01393975	1,37E+06	0,00646008	0,00E+00	0		
		0,02915106	1,43E+08	0,92511033	6,70E+06	0,04346799	3,34E+06	0,02163659	1,43E+06	0,00927731	7,83E+04	0,00050779		
		0,02253086	1,35E+08	0,94424051	4,08E+06	0,02846846	2,91E+06	0,02033893	9,78E+05	0,00683128	1,73E+04	0,00012082		

Table S1C. SCAT

			m+0		m+1		m+2		m+3		m+4		m+5		m+6	
	Condition	mean enrichment	Corrected area	fraction	Corrected area	fraction	Corrected area	fraction	Corrected area	fraction	Corrected area	fraction	Corrected area	fraction	Corrected area	fraction
lactate	4°C	0,18112	1,51E+09	0,80795736	2,28E+07	0,01224341	1,54E+07	0,00827294	3,20E+08	0,17152629						
		0,06044	1,63E+09	0,93352874	9,95E+06	0,00571387	1,16E+07	0,00666506	9,42E+07	0,05409233						
		0,16336	2,60E+09	0,8236615	4,13E+07	0,0131067	4,01E+07	0,01271932	4,74E+08	0,15051248						
		0,32667	3,21E+09	0,66403257	5,08E+07	0,01049758	3,34E+07	0,00689681	1,54E+09	0,31857303						
		0,19843	2,85E+09	0,78902968	4,44E+07	0,01231033	4,69E+07	0,01300348	6,70E+08	0,18565651						
	21°C	0,16999	1,28E+09	0,81937596	1,79E+07	0,01146216	1,40E+07	0,0089856	2,50E+08	0,16017627						
		0,19789	2,24E+09	0,79250649	2,87E+07	0,01016725	2,40E+07	0,00848746	5,33E+08	0,1888388						
		0,13526	2,08E+09	0,85550023	2,25E+07	0,0092716	2,23E+07	0,00916173	3,06E+08	0,12606645						
		0,10977	2,08E+09	0,87975185	2,78E+07	0,01176574	1,87E+07	0,00790207	2,38E+08	0,10058034						
		0,16459	1,93E+09	0,82525743	2,44E+07	0,01045018	2,23E+07	0,00955178	3,62E+08	0,15474061						
	30°C	0,16173	1,71E+09	0,81822654	4,07E+07	0,01943859	4,45E+07	0,02124143	2,95E+08	0,14109344						
		0,3016	1,52E+09	0,68349171	3,34E+07	0,01503657	3,26E+07	0,01466604	6,37E+08	0,28680568						
		0,08254	1,43E+09	0,90289527	2,35E+07	0,01483149	2,22E+07	0,01403162	1,08E+08	0,06824162						
		0,20165	1,82E+09	0,78730926	2,66E+07	0,01146812	2,36E+07	0,01018274	4,43E+08	0,19103988						
		0,2045	1,86E+09	0,78020842	3,73E+07	0,01562943	3,49E+07	0,01462729	4,52E+08	0,18953485						
pyruvate	4°C	0,09941	8,85E+06	0,8954477	7,63E+04	0,00771915	0,00E+00	0	9,57E+05	0,09683315						
		0,02619	2,33E+07	0,97178165	6,03E+04	0,00251163	2,52E+04	0,00104719	5,92E+05	0,02465953						
		0,11168	2,32E+07	0,87650148	3,49E+05	0,01317835	2,41E+05	0,00909472	2,68E+06	0,10122545						
		0,22897	3,58E+07	0,7602669	5,99E+05	0,0127235	3,22E+05	0,00682777	1,04E+07	0,22018183						
		0,1394	3,80E+07	0,84832584	5,78E+05	0,012923	4,91E+05	0,01096528	5,72E+06	0,12778588						
	21°C	0,0978	1,37E+07	0,90137494	1,60E+04	0,00105317	5,52E+03	0,00036293	1,48E+06	0,09720896						
		0,11997	2,11E+07	0,87609695	1,12E+05	0,00466205	5,97E+04	0,00247351	2,82E+06	0,1167675						
		0,11328	3,05E+07	0,87703889	3,58E+05	0,01030364	2,93E+05	0,00844326	3,62E+06	0,10421422						
		0,06805	2,12E+07	0,92621201	1,62E+05	0,00707352	6,98E+04	0,003053	1,46E+06	0,06366148						
		0,11087	1,99E+07	0,88300157	1,40E+05	0,0062115	1,34E+05	0,00597593	2,36E+06	0,104811						
	30°C	0,11084	1,87E+07	0,8776771	2,35E+05	0,01101352	2,64E+05	0,01240726	2,11E+06	0,09890212						
		0,23314	1,86E+07	0,75605848	2,59E+05	0,01053694	2,78E+05	0,01132201	5,46E+06	0,22208257						
		0,04896	1,82E+07	0,94293761	1,69E+05	0,00875574	1,31E+05	0,0067933	8,00E+05	0,04151334						
		0,13143	2,10E+07	0,86478182	7,69E+04	0,00315827	1,23E+05	0,00504873	3,09E+06	0,12701118						
		0,15798	2,43E+07	0,8313741	3,38E+05	0,01157993	2,56E+05	0,00877669	4,33E+06	0,14826928						
CisAco	4°C	0,0316	1,60E+07	0,89628479	7,23E+05	0,04045151	7,81E+05	0,04365348	2,97E+05	0,01660677	5,37E+04	0,00300345	0,00E+00	0	0,00E+00	0
		0,01314	2,21E+06	0,92648697	1,63E+05	0,06853714	1,11E+04	0,00463615	8,10E+02	0,00033974	0,00E+00	0	0,00E+00	0	0,00E+00	0
		0,02838	1,51E+07	0,90085947	7,81E+05	0,04671247	5,64E+05	0,0337226	3,13E+05	0,01870546	0,00E+00	0	0,00E+00	0	0,00E+00	0
		0,03735	1,61E+07	0,87457011	9,92E+05	0,05388766	8,65E+05	0,04700373	4,06E+05	0,02206954	4,30E+04	0,00233767	2,42E+03	0,00013128	0,00E+00	0
		0,03782	3,23E+07	0,87370841	2,10E+06	0,05682287	1,55E+06	0,04183022	8,91E+05	0,02409427	1,31E+05	0,00354423	0,00E+00	0	0,00E+00	0
	21°C	0,01323	4,69E+06	0,94781238	1,40E+05	0,02822642	1,07E+05	0,02164057	6,91E+03	0,00139718	4,57E+03	0,00092345	0,00E+00	0	0,00E+00	0
		0,04256	1,21E+07	0,85833748	8,14E+05	0,05759117	8,08E+05	0,05715769	3,42E+05	0,02417654	3,87E+04	0,00273712	0,00E+00	0	0,00E+00	0
		0,03138	1,59E+07	0,89726245	7,01E+05	0,03961128	7,42E+05	0,0419325	3,54E+05	0,02000255	2,00E+04	0,00113091	1,07E+03	6,0296E-05	0,00E+00	0
		0,02534	6,34E+06	0,90751812	2,87E+05	0,04105306	3,03E+05	0,04331586	5,67E+04	0,00811295	0,00E+00	0	0,00E+00	0	0,00E+00	0
		0,03335	1,10E+07	0,88444731	6,29E+05	0,05041571	5,71E+05	0,04575074	2,42E+05	0,01938624	0,00E+00	0	0,00E+00	0	0,00E+00	0
	30°C	0,05062	8,54E+06	0,83785941	5,95E+05	0,05842014	7,27E+05	0,07140403	2,73E+05	0,02675797	5,66E+04	0,00555845	0,00E+00	0	0,00E+00	0
		0,02846	5,51E+06	0,90788271	1,89E+05	0,03112199	2,69E+05	0,04426189	9,87E+04	0,0162683	0,00E+00	0	2,82E+03	0,00046511	0,00E+00	0
		0,01451	6,64E+06	0,94254144	2,11E+05	0,02995133	1,88E+05	0,02674066	0,00E+00	0	1,47E+03	0,00020926	3,92E+03	0,0005573	0,00E+00	0
		0,02973	7,69E+06	0,90251463	3,24E+05	0,0380171	3,54E+05	0,04157145	1,26E+05	0,01473395	2,40E+04	0,00281924	2,93E+03	0,00034364	0,00E+00	0
		0,03288	1,10E+07	0,89107981	5,43E+05	0,04414589	5,25E+05	0,04262987	2,55E+05	0,02068958	1,79E+04	0,00145485	0,00E+00	0	0,00E+00	0
Akg	4°C	0,04873	1,83E+07	0,86730559	8,90E+05	0,04222698	1,50E+06	0,07140263	3,75E+05	0,01781213	2,28E+04	0,0010833	3,57E+03	0,00016937		
		0,01656	2,83E+07	0,95298728	4,88E+05	0,01645406	7,58E+05	0,02556998	1,44E+05	0,00486002	0,00E+00	0	3,82E+03	0,00012866		
		0,04453	6,43E+07	0,87785893	3,08E+06	0,04201257	4,50E+06	0,06145409	1,25E+06	0,01713154	1,01E+05	0,00137504	1,23E+04	0,00016783		

		0,03091	4,51E+07	0,91211616	1,57E+06	0,03170644	2,30E+06	0,04649571	4,43E+05	0,00895877	3,26E+04	0,00065948	3,14E+03	6,3444E-05
		0,04938	6,52E+07	0,86626573	3,33E+06	0,04426198	5,14E+06	0,06825206	1,44E+06	0,01915059	1,26E+05	0,00167033	3,01E+04	0,00039931
	21°C	0,03594	1,73E+07	0,90582117	4,21E+05	0,02211425	1,15E+06	0,06029225	1,97E+05	0,01033388	2,23E+04	0,00117063	5,10E+03	0,00026783
		0,05546	2,88E+07	0,85460471	1,41E+06	0,04187589	2,72E+06	0,08068656	6,12E+05	0,01818113	1,26E+05	0,00375028	3,04E+04	0,00090143
		0,05847	6,74E+07	0,84257982	4,37E+06	0,05466244	6,09E+06	0,07618179	1,76E+06	0,0220613	2,74E+05	0,00342773	8,69E+04	0,00108692
		0,0447	5,33E+07	0,87873542	2,35E+06	0,03879448	4,06E+06	0,06687162	7,37E+05	0,01214367	1,68E+05	0,00276322	4,19E+04	0,00069159
		0,04059	4,15E+07	0,88724234	1,77E+06	0,0378918	2,84E+06	0,06067331	6,21E+05	0,01328147	3,15E+04	0,00067456	1,11E+04	0,00023652
	30°C	0,08158	3,45E+07	0,79495628	2,26E+06	0,05197764	5,04E+06	0,11604097	1,17E+06	0,02700203	3,16E+05	0,00728089	1,19E+05	0,00274219
		0,04641	4,76E+07	0,87728464	1,96E+06	0,03606636	3,68E+06	0,06786911	8,31E+05	0,0153113	1,64E+05	0,00301491	2,46E+04	0,00045367
		0,04715	2,90E+07	0,86871012	1,46E+06	0,04379865	2,42E+06	0,07240291	4,47E+05	0,01339898	4,93E+04	0,00147665	7,10E+03	0,00021268
		0,0605	2,66E+07	0,84858442	1,20E+06	0,03846599	2,64E+06	0,08449533	6,47E+05	0,02067055	1,84E+05	0,00588773	5,93E+04	0,00189598
		0,05201	4,92E+07	0,865866	2,19E+06	0,0385445	4,08E+06	0,07187457	1,05E+06	0,01849374	2,18E+05	0,00384328	7,82E+04	0,00137791
Gln	4°C	0,03351	1,13E+08	0,9136425	2,99E+06	0,02415643	5,60E+06	0,04524476	1,88E+06	0,0151777	1,89E+05	0,00152666	3,12E+04	0,00025196
		0,02117	1,55E+08	0,939312	4,01E+06	0,02431671	4,55E+06	0,02758138	1,45E+06	0,0087899	0,00E+00	0	0,00E+00	0
		0,05265	3,02E+08	0,85615984	1,86E+07	0,05274384	2,30E+07	0,06536262	8,35E+06	0,02367535	5,44E+05	0,00154257	1,82E+05	0,00051579
		0,03699	3,76E+08	0,90390448	1,30E+07	0,03126574	1,90E+07	0,04573348	6,36E+06	0,01530307	1,10E+06	0,00264949	4,75E+05	0,00114374
		0,0447	4,20E+08	0,88458536	1,62E+07	0,03402238	2,75E+07	0,05797997	9,85E+06	0,02074412	9,65E+05	0,00203232	3,02E+05	0,00063584
	21°C	0,03343	1,81E+08	0,91194453	6,04E+06	0,03047037	8,10E+06	0,04088283	2,53E+06	0,01276721	6,11E+05	0,00308424	1,69E+05	0,00085081
		0,03831	2,85E+08	0,9044488	8,25E+06	0,02613522	1,56E+07	0,04936392	4,72E+06	0,01497315	1,14E+06	0,00362226	4,60E+05	0,00145666
		0,04249	2,84E+08	0,88943809	1,10E+07	0,03462218	1,73E+07	0,05425341	5,80E+06	0,01819487	8,76E+05	0,0027442	2,38E+05	0,00074724
		0,04133	2,93E+08	0,89401724	1,04E+07	0,03181971	1,77E+07	0,053915	5,03E+06	0,01534935	1,16E+06	0,00354232	4,45E+05	0,00135637
		0,03621	3,07E+08	0,90679442	9,45E+06	0,02785684	1,55E+07	0,04577391	5,84E+06	0,01720996	6,08E+05	0,00179159	1,94E+05	0,00057328
	30°C	0,05521	1,40E+08	0,87119197	4,54E+06	0,02829099	1,08E+07	0,06757807	3,57E+06	0,02226553	1,22E+06	0,00757194	4,98E+05	0,0031015
		0,089	2,41E+08	0,79243444	1,45E+07	0,04752266	3,19E+07	0,10478173	1,15E+07	0,03789306	3,83E+06	0,01258304	1,46E+06	0,00478508
		0,04146	1,50E+08	0,89053029	6,01E+06	0,03577433	9,17E+06	0,05458832	2,50E+06	0,0149166	5,64E+05	0,00335908	1,40E+05	0,00083138
		0,03739	1,39E+08	0,90978366	3,32E+06	0,02173359	7,32E+06	0,04795788	2,22E+06	0,01455532	6,45E+05	0,00423048	2,65E+05	0,00173906
		0,04405	1,25E+08	0,89377001	3,39E+06	0,02425107	8,07E+06	0,05769863	2,52E+06	0,01804131	6,58E+05	0,00470602	2,14E+05	0,00153296
Glu	4°C	0,01725	4,80E+08	0,95403944	7,20E+06	0,01430798	1,20E+07	0,0237895	3,60E+06	0,00716545	3,19E+05	0,00063356	3,22E+04	6,4063E-05
		0,01157	3,74E+08	0,96706863	4,69E+06	0,01213307	6,48E+06	0,01677029	1,53E+06	0,00395844	2,69E+04	6,9573E-05	0,00E+00	0
		0,02813	4,69E+08	0,92284616	1,30E+07	0,02564471	2,04E+07	0,04011065	5,50E+06	0,01081856	2,87E+05	0,00056502	7,57E+03	1,49E-05
		0,02896	4,12E+08	0,91985855	1,25E+07	0,02790267	1,87E+07	0,04180579	3,94E+06	0,00878886	5,79E+05	0,00129303	1,57E+05	0,0003511
		0,02803	4,84E+08	0,92074429	1,49E+07	0,02838672	2,19E+07	0,04162484	4,45E+06	0,00847334	3,87E+05	0,00073622	1,82E+04	3,4597E-05
	21°C	0,02301	3,89E+08	0,93732457	7,99E+06	0,01924171	1,50E+07	0,03610338	2,43E+06	0,00584087	5,67E+05	0,0013647	5,18E+04	0,00012477
		0,03087	4,31E+08	0,91766074	9,82E+06	0,0208818	2,50E+07	0,05314737	3,01E+06	0,00639501	7,43E+05	0,00158085	1,57E+05	0,00033422
		0,02209	4,25E+08	0,9396741	7,65E+06	0,01692537	1,69E+07	0,03743248	2,35E+06	0,00520908	3,42E+05	0,00075614	1,28E+03	2,8274E-06
		0,02614	3,58E+08	0,92977557	7,63E+06	0,01984814	1,63E+07	0,04235303	2,38E+06	0,00618166	6,12E+05	0,00159102	9,64E+04	0,00025059
		0,02458	4,22E+08	0,93399397	7,65E+06	0,01692011	1,90E+07	0,04200717	2,87E+06	0,00634332	3,30E+05	0,00072902	2,90E+03	6,4119E-06
	30°C	0,03685	4,78E+08	0,90948731	9,19E+06	0,0174727	3,03E+07	0,05769844	5,80E+06	0,01103108	1,71E+06	0,0032431	5,61E+05	0,00106737
		0,03102	3,68E+08	0,91968291	7,09E+06	0,017721	2,13E+07	0,05318765	2,85E+06	0,00712138	7,21E+05	0,00180323	1,94E+05	0,00048383
		0,02383	3,57E+08	0,93590831	5,99E+06	0,01568593	1,63E+07	0,04271908	1,82E+06	0,00475604	3,36E+05	0,00088053	1,91E+04	5,0117E-05
		0,02613	3,55E+08	0,93102983	8,50E+06	0,02228046	1,35E+07	0,0354146	3,17E+06	0,00829345	8,51E+05	0,00222928	2,87E+05	0,00075239
		0,02002	5,84E+08	0,94667812	8,74E+06	0,01417217	2,04E+07	0,03306449	2,94E+06	0,00476024	6,75E+05	0,00109424	1,42E+05	0,00023072
Succ	4°C	0,03573	5,57E+08	0,91352828	2,45E+07	0,04023444	2,25E+07	0,03691727	5,13E+06	0,00842405	5,46E+05	0,00089597		
		0,01219	4,86E+07	0,96696957	9,30E+05	0,01849556	6,71E+05	0,01333442	6,04E+04	0,00120045	0,00E+00	0		
		0,05713	4,71E+08	0,86015401	3,72E+07	0,06797691	3,10E+07	0,05672033	7,38E+06	0,01349269	9,06E+05	0,00165606		
		0,05411	4,24E+08	0,86580927	3,32E+07	0,06782731	2,57E+07	0,05240296	5,88E+06	0,01201958	9,50E+05	0,00194087		
		0,0573	4,74E+08	0,86281899	3,48E+07	0,06323664	3,18E+07	0,05783205	7,78E+06	0,01416097	1,07E+06	0,00195135		
	21°C	0,04497	4,24E+08	0,89211411	2,33E+07	0,04910616	2,27E+07	0,04765629	4,29E+06	0,00902165	9,99E+05	0,00210179		
		0,06105	5,10E+08	0,85033341	4,41E+07	0,07358001	3,61E+07	0,06017033	8,02E+06	0,01337871	1,52E+06	0,00253755		
		0,04778	3,65E+08	0,88161679	2,37E+07	0,05718916	2,12E+07	0,05132246	3,40E+06	0,00821876	6,84E+05	0,00165283		
		0,04831	4,80E+08	0,87896033	3,34E+07	0,06120848	2,69E+07	0,04925126	4,81E+06	0,00879675	9,74E+05	0,00178318		
		0,04741	3,79E+08	0,88211332	2,42E+07	0,05631625	2,26E+07	0,05266037	3,28E+06	0,00764193	5,45E+05	0,00126812		
	30°C	0,07385	4,12E+08	0,83337111	3,26E+07	0,06587834	3,84E+07	0,07767789	8,96E+06	0,01813158	2,44E+06	0,00494108		

		0,07623	3,09E+08	0,8293171	2,39E+07	0,06423714	3,16E+07	0,08470874	5,85E+06	0,01570024	2,25E+06	0,00603677
		0,04112	3,06E+08	0,89843051	1,65E+07	0,04825309	1,54E+07	0,04507471	2,35E+06	0,00689224	4,60E+05	0,00134945
		0,06638	3,58E+08	0,84550206	2,95E+07	0,06962107	2,63E+07	0,06224609	8,09E+06	0,01911815	1,49E+06	0,00351262
		0,04907	3,84E+08	0,88714764	2,01E+07	0,04637344	2,25E+07	0,05193543	5,26E+06	0,01215309	1,03E+06	0,0023904
Asp	4°C	0,00664	4,14E+08	0,98651834	1,76E+06	0,00418484	2,33E+06	0,00554156	1,57E+06	0,00374096	6,00E+03	1,43E-05
		0,0048	2,50E+08	0,98845888	1,38E+06	0,00544763	1,15E+06	0,00454079	3,93E+05	0,0015527	0,00E+00	0
		0,01747	3,00E+08	0,95993967	5,59E+06	0,01788691	4,72E+06	0,01511538	2,03E+06	0,00648776	1,78E+05	0,00057027
		0,02205	2,68E+08	0,95099528	5,54E+06	0,01966798	5,66E+06	0,02009234	2,43E+06	0,00863705	1,71E+05	0,00060735
		0,01832	2,92E+08	0,95962698	4,36E+06	0,01429715	5,98E+06	0,01964062	1,84E+06	0,00604896	1,18E+05	0,00038629
	21°C	0,01535	2,97E+08	0,96490591	4,72E+06	0,01534548	4,09E+06	0,01329582	1,95E+06	0,0063409	3,44E+04	0,0001119
		0,01987	4,34E+08	0,95504351	7,06E+06	0,01554065	1,12E+07	0,02470491	1,95E+06	0,00429838	1,88E+05	0,00041255
		0,01685	3,59E+08	0,96268835	5,03E+06	0,01348538	6,69E+06	0,01793041	2,07E+06	0,00554885	1,29E+05	0,00034702
		0,01646	2,34E+08	0,96135078	3,97E+06	0,0163245	4,31E+06	0,01770294	1,06E+06	0,00436276	6,31E+04	0,00025902
		0,01663	3,60E+08	0,96282887	4,98E+06	0,01330958	6,99E+06	0,01868755	1,82E+06	0,00485505	1,19E+05	0,00031895
	30°C	0,01868	3,44E+08	0,96070017	4,52E+06	0,01261821	6,67E+06	0,01861891	2,65E+06	0,00737939	2,45E+05	0,00068331
		0,0197	2,82E+08	0,95568101	5,25E+06	0,01778623	5,65E+06	0,01911448	2,03E+06	0,0068724	1,61E+05	0,00054587
		0,01141	2,96E+08	0,97545026	1,92E+06	0,00632413	4,67E+06	0,01538085	8,63E+05	0,00284476	0,00E+00	0
		0,01282	2,02E+08	0,97285583	2,12E+06	0,01025078	2,01E+06	0,00971574	1,48E+06	0,00712179	1,16E+04	5,5859E-05
		0,0098	4,62E+08	0,97965955	2,71E+06	0,0057399	4,90E+06	0,01039796	1,95E+06	0,00413546	3,17E+04	6,7127E-05

Table S1D. Liver

			m+0		m+1		m+2		m+3		m+4		m+5		m+6	
	Condition	mean enrichment	Corrected area	fraction	Corrected area	fraction	Corrected area	fraction	Corrected area	fraction	Corrected area	fraction	Corrected area	fraction	Corrected area	fraction
lactate	4°C	0,11122912	2,99E+09	0,86577916	8,10E+07	0,02347604	7,60E+07	0,02202309	3,06E+08	0,08872171						
		0,0566769	3,70E+09	0,92719115	6,08E+07	0,01522047	7,17E+07	0,01795491	1,58E+08	0,03963347						
		0,20092398	1,83E+09	0,77153758	6,35E+07	0,02681206	6,86E+07	0,02899121	4,09E+08	0,17265915						
		0,08039782	3,63E+09	0,89923119	8,70E+07	0,02153962	7,28E+07	0,01803372	2,47E+08	0,06119546						
		0,30180731	1,33E+09	0,66734561	6,12E+07	0,03071361	6,20E+07	0,03111402	5,40E+08	0,27082676						
	21°C	0,12472942	4,49E+09	0,84723084	1,52E+08	0,02860772	1,42E+08	0,02690378	5,15E+08	0,09725765						
		0,14301582	3,61E+09	0,83253262	1,12E+08	0,02580018	9,42E+07	0,02175431	5,19E+08	0,11991289						
		0,09642806	4,48E+09	0,88169113	1,12E+08	0,02213078	1,09E+08	0,02138088	3,80E+08	0,07479721						
		0,05865332	3,81E+09	0,91929716	1,00E+08	0,02425348	7,31E+07	0,01764159	1,61E+08	0,03880777						
		0,08223185	2,03E+08	0,89667409	5,00E+06	0,02212383	4,30E+06	0,01903452	1,40E+07	0,06216756						
	30°C	0,12710862	3,79E+09	0,82941175	1,93E+08	0,04220616	2,11E+08	0,04602655	3,77E+08	0,08235554						
		0,17262611	1,03E+09	0,79689948	3,66E+07	0,02830081	4,50E+07	0,0348216	1,81E+08	0,13997811						
		0,05853498	3,68E+08	0,91588709	1,03E+07	0,02563949	1,02E+07	0,02545483	1,33E+07	0,03301859						
		0,16915472	5,36E+09	0,79720214	2,23E+08	0,03321583	2,32E+08	0,03449779	9,09E+08	0,13508425						
		0,13163817	2,91E+09	0,83401143	1,12E+08	0,03223001	1,34E+08	0,03859119	3,32E+08	0,09516738						
pyruvate	4°C	0,06700793	1,12E+07	0,9235951	1,19E+05	0,00978562	1,04E+05	0,00861966	7,03E+05	0,05799962						
		0,029491	1,76E+07	0,96216253	1,46E+05	0,00797782	1,66E+05	0,00908376	3,80E+05	0,02077589						
		0,0978384	6,74E+06	0,88763544	1,15E+05	0,01509739	1,02E+05	0,01338371	6,37E+05	0,08388346						
		0,06269688	1,65E+07	0,92513804	2,38E+05	0,01334605	1,75E+05	0,00980313	9,21E+05	0,05171278						
		0,14912131	6,17E+06	0,83604783	1,15E+05	0,01555606	9,87E+04	0,01338044	9,96E+05	0,13501566						
	21°C	0,10129751	1,94E+07	0,88369444	3,35E+05	0,01523054	3,20E+05	0,01456307	1,90E+06	0,08651195						
		0,10228775	1,25E+07	0,88436744	2,03E+05	0,01431069	1,61E+05	0,01141305	1,27E+06	0,08990882						
		0,06809021	2,02E+07	0,91886147	2,82E+05	0,0128543	2,95E+05	0,01343637	1,20E+06	0,05484786						
		0,03237691	1,30E+07	0,95750242	1,55E+05	0,01145499	1,01E+05	0,00745203	3,20E+05	0,02359056						
	30°C	0,10531171	1,60E+07	0,86485618	5,54E+05	0,03004214	5,43E+05	0,02941206	1,40E+06	0,07568963						
		0,0986253	3,44E+06	0,89614319	1,07E+04	0,00277636	3,89E+04	0,01014181	3,49E+05	0,09093864						
		0,13393367	2,42E+07	0,84214058	6,75E+05	0,02351844	7,10E+05	0,02474034	3,15E+06	0,10960064						
		0,09686804	8,95E+06	0,88486279	1,67E+05	0,01650015	2,21E+05	0,02180723	7,77E+05	0,07682983						
Glucose	4°C	0,02521414	4,46E+08	0,93498801	1,15E+07	0,02415495	3,98E+06	0,00835127	1,18E+07	0,02473258	1,78E+06	0,00372657	1,41E+06	0,00295641	5,20E+05	0,00109022
		0,02716663	5,97E+08	0,92870008	1,26E+07	0,01954165	1,32E+07	0,02049055	1,66E+07	0,02583796	1,76E+06	0,00273746	1,38E+06	0,00214048	3,55E+05	0,00055182
		0,07883367	2,57E+08	0,7940182	2,15E+07	0,06632128	1,62E+07	0,05010108	2,18E+07	0,06736149	3,35E+06	0,01035561	2,62E+06	0,00808239	1,22E+06	0,00375995
		0,01254604	7,86E+08	0,96475498	1,47E+07	0,0180483	0,00E+00	0	1,10E+07	0,01347826	1,73E+06	0,00212161	1,04E+06	0,00127431	2,63E+05	0,00032253
		0,09975475	2,12E+08	0,73864033	2,10E+07	0,07317392	2,37E+07	0,08249353	2,30E+07	0,08003563	3,34E+06	0,01163076	2,99E+06	0,01041741	1,04E+06	0,00360843
	21°C	0,00783108	5,57E+08	0,97672347	7,00E+06	0,01227473	0,00E+00	0	5,48E+06	0,00961333	6,19E+05	0,00108555	1,64E+05	0,00028792	8,55E+03	1,50E-05
		0,00976028	6,47E+08	0,97199213	9,42E+06	0,01415406	0,00E+00	0	7,73E+06	0,01160913	1,10E+06	0,00165885	3,79E+05	0,00057011	1,05E+04	1,57E-05
		0,01086877	8,07E+08	0,97455914	6,43E+06	0,00776425	0,00E+00	0	1,21E+07	0,01463783	1,53E+06	0,00184327	8,38E+05	0,00101126	1,53E+05	0,00018425
		0,00222977	6,91E+08	0,99560663	0,00E+00	0	0,00E+00	0	2,91E+06	0,00419483	1,38E+05	0,00019854	0,00E+00	0	0,00E+00	0
		0,01617205	2,28E+07	0,95068236	4,86E+05	0,02030946	2,79E+05	0,01166157	3,93E+05	0,01642961	1,35E+04	0,00056357	6,32E+03	0,00026395	2,14E+03	8,95E-05
	30°C	0,01711575	4,78E+08	0,95576052	7,66E+06	0,01532374	2,68E+06	0,00536561	9,85E+06	0,01971558	1,06E+06	0,00211513	6,42E+05	0,00128426	2,17E+05	0,00043516
		0,01991329	1,48E+08	0,95657844	7,48E+05	0,00481972	1,70E+06	0,01094022	3,34E+06	0,0214948	5,13E+05	0,00330468	3,25E+05	0,0020964	1,19E+05	0,00076575
		0,00489911	4,80E+07	0,98840672	1,34E+05	0,00276986	0,00E+00	0	4,25E+05	0,00874614	0,00E+00	0	3,75E+03	7,73E-05	0,00E+00	0
		0,00876997	9,08E+08	0,98034942	4,66E+06	0,00502586	4,10E+05	0,00044257	1,05E+07	0,01135449	1,56E+06	0,00168889	8,73E+05	0,00094275	1,82E+05	0,000196
		0,01409748	3,89E+08	0,96224457	6,11E+06	0,01510639	1,55E+06	0,00383331	6,16E+06	0,01522307	8,80E+05	0,00217638	4,29E+05	0,00106059	1,44E+05	0,00035569
CisAco	4°C	0,07368181	1,87E+07	0,74557138	2,84E+06	0,11333371	2,37E+06	0,09452758	1,17E+06	0,04656733	0,00E+00	0	0,00E+00	0	0,00E+00	0
		0,04621959	2,64E+07	0,84963574	1,80E+06	0,05805211	1,79E+06	0,05767102	1,08E+06	0,03464114	0,00E+00	0	0,00E+00	0	0,00E+00	0
		0,08208919	2,40E+07	0,72383886	3,98E+06	0,12016654	3,20E+06	0,09666828	1,93E+06	0,05827323	3,49E+04	0,00105308	0,00E+00	0	0,00E+00	0
		0,07326589	2,10E+07	0,73750595	3,64E+06	0,12775127	2,63E+06	0,09238424	1,21E+06	0,04235854	0,00E+00	0	0,00E+00	0	0,00E+00	0
		0,06919733	2,23E+07	0,7765643	2,48E+06	0,08645578	2,36E+06	0,08221156	1,57E+06	0,05476836	0,00E+00	0	0,00E+00	0	0,00E+00	0
	21°C	0,08736754	2,00E+07	0,71104836	3,14E+06	0,11163505	3,50E+06	0,12449142	1,34E+06	0,04771333	1,44E+05	0,00511184	0,00E+00	0	0,00E+00	0
		0,09064424	1,21E+07	0,69772871	2,16E+06	0,12449855	2,05E+06	0,11803486	9,65E+05	0,05565433	7,08E+04	0,00408355	0,00E+00	0	0,00E+00	0
		0,08006374	2,95E+07	0,72109436	5,22E+06	0,12745493	4,19E+06	0,10221976	1,98E+06	0,0484358	3,26E+04	0,00079515	0,00E+00	0	0,00E+00	0
		0,0607648	1,44E+07	0,78254038	1,84E+06	0,09993234	1,62E+06	0,08792537	5,45E+05	0,0296019	0,00E+00	0	0,00E+00	0	0,00E+00	0
		0,0328004	8,00E+05	0,86204783	8,00E+04	0,08618539	4,15E+04	0,04468333	6,57E+03	0,00708345	0,00E+00	0	0,00E+00	0	0,00E+00	0
	30°C	0,09928893	2,25E+07	0,67680794	4,46E+06	0,13409059	3,89E+06	0,11697322	2,02E+06	0,06081645	3,76E+05	0,0113118	0,00E+00	0	0,00E+00	0
		0,08117604	9,35E+06	0,73636147	1,39E+06	0,10943866	1,11E+06	0,08772796	8,10E+05	0,063726	3,49E+04	0,00274591	0,00E+00	0	0,00E+00	0
		0,03299416	1,13E+06	0,84648215	1,47E+05	0,10998314	5,70E+04	0,04262233	1,22E+03	0,00091238	0,00E+00	0	0,00E+00	0	0,00E+00	0
		0,08506017	1,61E+07	0,73103578	2,35E+06	0,1065129	2,06E+06	0,09341041	1,31E+06	0,05936586	2,08E+05	0,0094455	5,06E+03	0,00022955	0,00E+00	0

		0,08643084	1,81E+07	0,72373308	2,76E+06	0,11019222	2,43E+06	0,09730646	1,53E+06	0,06129307	1,87E+05	0,00747517	0,00E+00	0	0,00E+00	0
		0,07040765	2,83E+07	0,80713769	2,22E+06	0,06349264	3,54E+06	0,10109104	9,37E+05	0,02675098	5,35E+04	0,00152765	0,00E+00	0		
		0,04671186	5,10E+07	0,87331379	2,27E+06	0,03889452	4,01E+06	0,06871031	1,11E+06	0,01908139	0,00E+00	0	0,00E+00	0		
	4°C	0,06787577	3,65E+07	0,81525539	2,68E+06	0,05998496	4,25E+06	0,09488509	1,34E+06	0,02987456	0,00E+00	0	0,00E+00	0		
		0,06451912	2,97E+07	0,82138893	2,24E+06	0,06217912	3,23E+06	0,08938227	9,63E+05	0,02668045	8,50E+03	0,00023553	4,83E+03	0,0001337		
		0,06136113	2,85E+07	0,83856383	1,56E+06	0,04582508	2,92E+06	0,08585273	1,01E+06	0,02975837	0,00E+00	0	0,00E+00	0		
		0,10122145	3,18E+07	0,73734066	3,18E+06	0,07363606	6,23E+06	0,14441297	1,54E+06	0,03566932	3,48E+05	0,00806765	3,77E+04	0,00087333		
		0,08596424	2,40E+07	0,77933864	1,72E+06	0,05599239	3,89E+06	0,12648443	9,97E+05	0,0323948	1,62E+05	0,0052731	1,59E+04	0,00051663		
Akg	21°C	0,07730047	3,98E+07	0,79161657	3,22E+06	0,0640084	5,61E+06	0,11157639	1,61E+06	0,03196465	3,63E+04	0,0007227	5,60E+03	0,00011128		
		0,06663919	2,31E+07	0,81584303	1,76E+06	0,06217176	2,78E+06	0,09811306	5,94E+05	0,02099291	7,29E+04	0,00257684	8,56E+03	0,0003024		
		0,03091311	1,63E+06	0,91286828	3,77E+04	0,02112201	1,15E+05	0,06458558	2,54E+03	0,00142413	0,00E+00	0	0,00E+00	0		
		0,10566851	3,72E+07	0,72501388	4,05E+06	0,07892213	7,46E+06	0,14554234	2,28E+06	0,04453997	2,66E+05	0,00519261	4,05E+04	0,00078908		
		0,07933699	1,22E+07	0,79581364	7,70E+05	0,05012094	1,78E+06	0,11608865	5,80E+05	0,03774857	0,00E+00	0	3,51E+03	0,0002282		
	30°C	0,05081143	2,93E+06	0,85802654	1,67E+05	0,04889266	2,55E+05	0,07481061	5,98E+04	0,01753752	2,50E+03	0,00073267	0,00E+00	0		
		0,08714094	3,41E+07	0,78483104	2,22E+06	0,05112209	5,18E+06	0,11909339	1,55E+06	0,03557121	3,14E+05	0,00722911	9,36E+04	0,00215315		
		0,09446883	3,44E+07	0,74963507	3,37E+06	0,07346304	6,14E+06	0,13385112	1,89E+06	0,04124749	7,25E+04	0,00157998	1,02E+04	0,0002233		
		0,09601226	8,32E+08	0,75192599	8,24E+07	0,074553	1,35E+08	0,12250155	5,00E+07	0,04522492	4,58E+06	0,00414227	1,83E+06	0,00165227		
		0,06126852	1,78E+09	0,84131121	8,97E+07	0,04237132	1,81E+08	0,08532792	6,49E+07	0,03064273	7,34E+05	0,00034681	0,00E+00	0		
	4°C	0,08545932	9,64E+08	0,78202862	7,41E+07	0,06008291	1,36E+08	0,11026607	5,51E+07	0,04469972	2,50E+06	0,00203103	1,10E+06	0,00089166		
		0,07790058	8,10E+08	0,7954873	6,64E+07	0,06520741	1,01E+08	0,09874872	3,72E+07	0,03654192	2,95E+06	0,00290095	1,13E+06	0,0011137		
		0,07960052	9,61E+08	0,79832783	6,48E+07	0,05377528	1,25E+08	0,10352299	4,96E+07	0,04121719	2,71E+06	0,00225377	1,09E+06	0,00090294		
		0,11353297	7,24E+08	0,73136121	6,52E+07	0,06586683	1,34E+08	0,13483725	4,57E+07	0,04611986	1,52E+07	0,01531027	6,44E+06	0,00650457		
		0,10982753	6,83E+08	0,73700624	6,23E+07	0,06718582	1,20E+08	0,12910824	4,50E+07	0,04857259	1,17E+07	0,01261793	5,11E+06	0,00550917		
Gln	21°C	0,08619374	1,19E+09	0,77604834	1,05E+08	0,06829711	1,69E+08	0,11027619	6,24E+07	0,04071739	5,11E+06	0,0033378	2,03E+06	0,00132316		
		0,1032202	7,68E+08	0,74048824	8,08E+07	0,0779493	1,31E+08	0,12647003	4,11E+07	0,03965548	1,13E+07	0,01093956	4,66E+06	0,00449739		
		0,08853484	6,94E+07	0,76763185	6,37E+06	0,07046139	1,04E+07	0,11551293	4,02E+06	0,04448156	1,64E+05	0,00181912	8,42E+03	9,32E-05		
		0,12073191	6,14E+08	0,70886721	6,40E+07	0,07378698	1,24E+08	0,14248711	5,16E+07	0,05951917	8,98E+06	0,01035682	4,32E+06	0,00498271		
		0,10367029	1,83E+08	0,742022	1,61E+07	0,06563363	3,24E+07	0,13162451	1,36E+07	0,05514272	9,46E+05	0,00384508	4,26E+05	0,00173206		
	30°C	0,07897779	9,04E+07	0,79128621	7,54E+06	0,06602457	1,18E+07	0,10355864	4,06E+06	0,03559276	3,11E+05	0,00272024	9,34E+04	0,00081757		
		0,11607253	9,82E+08	0,73512669	7,44E+07	0,05568746	1,79E+08	0,13365361	6,99E+07	0,05229115	2,10E+07	0,01571091	1,01E+07	0,00753018		
		0,09724198	6,37E+08	0,7610922	5,10E+07	0,06086418	1,00E+08	0,11998965	4,15E+07	0,04959225	4,79E+06	0,00571895	2,30E+06	0,00274278		
		0,07782825	4,47E+08	0,7908215	3,84E+07	0,06788399	5,92E+07	0,10463625	1,97E+07	0,03479555	9,70E+05	0,00171544	8,33E+04	0,00014727		
		0,06679269	1,22E+09	0,82584176	7,00E+07	0,04732122	1,39E+08	0,09386881	4,88E+07	0,03296821	0,00E+00	0	0,00E+00	0		
	4°C	0,08863068	1,09E+09	0,76745616	9,85E+07	0,06959238	1,64E+08	0,11595123	6,57E+07	0,04638206	8,19E+05	0,00057849	5,62E+04	3,97E-05		
		0,06574205	4,09E+08	0,8184862	3,24E+07	0,06476097	4,37E+07	0,0872951	1,43E+07	0,02854994	4,15E+05	0,0008297	3,91E+04	7,81E-05		
		0,08306172	1,14E+09	0,78263181	9,29E+07	0,06406981	1,59E+08	0,10958065	6,22E+07	0,04290993	1,00E+06	0,00069134	1,69E+05	0,00011647		
		0,10338922	3,92E+08	0,74341264	3,62E+07	0,06863967	7,07E+07	0,13410511	2,05E+07	0,03891545	5,95E+06	0,0112858	1,92E+06	0,00364133		
		0,09091952	2,90E+08	0,77022572	2,38E+07	0,06322053	4,55E+07	0,1207492	1,36E+07	0,03593886	2,74E+06	0,00726641	9,80E+05	0,00259929		
Glu	21°C	0,08213167	9,23E+08	0,77984144	8,57E+07	0,07236573	1,28E+08	0,10798242	4,42E+07	0,03733252	2,44E+06	0,00205925	4,96E+05	0,00041864		
		0,07695942	3,66E+08	0,79512155	3,11E+07	0,06769111	4,77E+07	0,10377634	1,19E+07	0,02582995	2,69E+06	0,0058418	8,00E+05	0,00173925		
		0,07932822	6,99E+07	0,78896653	5,67E+06	0,06397453	9,62E+06	0,10855716	3,41E+06	0,03847834	0,00E+00	0	2,08E+03	2,35E-05		
		0,10594965	4,20E+08	0,73086247	4,19E+07	0,07303779	8,12E+07	0,14138211	2,70E+07	0,04704526	3,19E+06	0,00555143	1,22E+06	0,00212094		
		0,0895286	2,79E+08	0,77207731	2,19E+07	0,06071226	4,23E+07	0,1170474	1,74E+07	0,04806691	6,67E+05	0,00184539	9,06E+04	0,00025073		
	30°C	0,06774739	8,34E+07	0,81676319	5,75E+06	0,05632313	1,01E+07	0,09843565	2,90E+06	0,02836958	1,11E+04	0,00010846	0,00E+00	0		
		0,08983809	4,31E+08	0,78033697	2,81E+07	0,0508518	6,77E+07	0,1226821	1,92E+07	0,03472106	4,54E+06	0,00822908	1,76E+06	0,00317899		
		0,08394595	2,52E+08	0,78308571	1,92E+07	0,05970388	3,70E+07	0,11508734	1,26E+07	0,03905956	8,51E+05	0,00264502	1,35E+05	0,00041848		
		0,11362909	9,21E+08	0,73013498	1,49E+08	0,11835553	1,54E+08	0,12170357	3,34E+07	0,02647	4,21E+06	0,00333593				
		0,09587037	1,30E+09	0,77835793	1,41E+08	0,08459246	1,88E+08	0,11278584	3,96E+07	0,02373774	8,78E+05	0,00052604				
	4°C	0,12050561	9,03E+08	0,72615268	1,34E+08	0,10756787	1,59E+08	0,1282067	4,26E+07	0,03424986	4,75E+06	0,0038229				
		0,10320188	8,95E+08	0,74347259	1,48E+08	0,12310108	1,36E+08	0,11266924	2,25E+07	0,0186604	2,52E+06	0,0020967				
		0,12129229	8,68E+08	0,72598096	1,31E+08	0,10928255	1,46E+08	0,12250554	4,55E+07	0,03804827	5,00E+06	0,00418267				
		0,14997027	8,35E+08	0,66180179	1,75E+08	0,13869062	1,89E+08	0,15017172	4,60E+07	0,03649641	1,62E+07	0,01283945				
		0,13545613	7,60E+08	0,68284078	1,58E+08	0,14164198	1,51E+08	0,13564385	3,41E+07	0,03059869	1,03E+07	0,00927469				
Succ	21°C	0,10908179	1,13E+09	0,73993506	1,72E+08	0,11277614	1,85E+08	0,1216076	3,41E+07	0,02238896	5,02E+06	0,00329223				
		0,09332843	7,23E+08	0,75536045	1,23E+08	0,12884388	1,05E+08	0,10935643	0,00E+00	0	6,16E+06	0,00643924				
		0,11440552	1,94E+08	0,72542594	3,23E+07	0,12088864	3,39E+07	0,12675447	6,55E+06	0,02449928	6,50E+05	0,00243166				
		0,15988223	8,67E+08	0,64068234	1,89E+08	0,1393262	2,29E+08	0,16915649	5,61E+07	0,04145014	1,27E+07	0,00938483				
		0,14392325	3,89E+08	0,68761919	6,14E+07	0,10851391	8,54E+07	0,15092771	2,63E+07	0,04643305	3,68E+06	0,00650613				
	30°C	0,10319851	2,19E+08	0,75244295	3,12E+07	0,10741719	3,41E+07	0,11726921	6,00E+06	0,02064418	6,47E+05	0,00222647				
		0,1666851	9,19E+08	0,63655056	1,99E+08	0,13769808	2,35E+08	0,16317938	6,87E+07	0,04760432	2,16E+07	0,01496765				
		0,15073322	7,11E+08	0,66203354	1,37E+08	0,12736808	1,75E+08	0,16265456	4,46E+07	0,04151958	6,90E+06	0,00642423				
Asp	4°C	0,04135198	1,90E+08	0,90527284	8,29E+06	0,03961779	8,36E+06	0,03992066	3,10E+06	0,01480604	8,01E+04	0,00038267				
		0,03360351	2,74E+08	0,9209519	9,94E+06	0,03336604	1,07E+07	0,03599816	2,89E+06	0,0096839	0,00E+00	0				

		0,0501097	1,97E+08	0,88838784	9,75E+06	0,04407321	1,03E+07	0,04656226	4,57E+06	0,02066568	6,88E+04	0,00031101
		0,03549238	1,60E+08	0,915297	6,81E+06	0,03896833	5,98E+06	0,03420282	2,02E+06	0,01153185	0,00E+00	0
		0,06115801	2,49E+08	0,86716724	1,48E+07	0,05143698	1,49E+07	0,05193538	8,19E+06	0,0285173	2,71E+05	0,00094311
		0,05940573	1,72E+08	0,86638623	1,08E+07	0,05414378	1,15E+07	0,05758741	3,82E+06	0,01922602	5,28E+05	0,00265656
		0,052399	1,53E+08	0,8802533	8,57E+06	0,04927831	9,13E+06	0,05250832	2,88E+06	0,01653924	2,47E+05	0,00142082
	21°C	0,03904275	2,48E+08	0,91054383	9,97E+06	0,03658219	1,06E+07	0,03903314	3,77E+06	0,01384084	0,00E+00	0
		0,04646985	1,86E+08	0,88841478	1,04E+07	0,04976182	1,04E+07	0,04983462	2,40E+06	0,01150679	1,01E+05	0,00048199
		0,03893987	2,71E+07	0,9134977	9,47E+05	0,03196907	1,18E+06	0,03980929	4,36E+05	0,01472394	0,00E+00	0
		0,08755409	2,02E+08	0,80319706	1,99E+07	0,07948433	2,13E+07	0,08490823	7,21E+06	0,0287259	9,24E+05	0,00368447
		0,0516016	1,03E+08	0,88866489	4,85E+06	0,04206265	5,32E+06	0,04608756	2,37E+06	0,02057096	3,02E+05	0,00261394
	30°C	0,03504179	4,22E+07	0,92064165	1,34E+06	0,02921911	1,81E+06	0,03946969	4,89E+05	0,01066955	0,00E+00	0
		0,08164354	2,19E+08	0,82528058	1,73E+07	0,06506647	1,93E+07	0,07275661	8,38E+06	0,03159087	1,41E+06	0,00530547
		0,0570015	1,24E+08	0,87202258	6,90E+06	0,04830971	8,47E+06	0,05932021	2,90E+06	0,02033415	1,91E+03	1,34E-05

Table S1E. Kidney

			m+0		m+1		m+2		m+3		m+4		m+5		m+6	
	Condition	mean enrichment	Corrected area	fraction	Corrected area	fraction	Corrected area	fraction	Corrected area	fraction	Corrected area	fraction	Corrected area	fraction	Corrected area	fraction
lactate	4°C	0,10384723	2,70E+09	0,86378623	1,03E+08	0,03306466	9,69E+07	0,0309703	2,26E+08	0,07217881						
		0,09028296	2,81E+09	0,88814222	6,54E+07	0,02066439	7,40E+07	0,0233957	2,14E+08	0,06779769						
		0,1404014	2,67E+09	0,8170656	1,37E+08	0,04188408	1,43E+08	0,04383085	3,18E+08	0,09721947						
		0,09424102	2,74E+09	0,8743863	9,83E+07	0,03138881	9,82E+07	0,03134042	1,97E+08	0,06288447						
		0,15957104	3,12E+09	0,80400201	1,38E+08	0,03557862	1,48E+08	0,0381236	4,74E+08	0,12229577						
	21°C	0,10070409	2,14E+09	0,87173843	7,22E+07	0,02942469	5,85E+07	0,02382308	1,84E+08	0,07501381						
		0,11444998	3,34E+09	0,85721417	1,16E+08	0,02981745	9,88E+07	0,02537264	3,41E+08	0,08759574						
		0,1156953	2,78E+09	0,85254402	1,05E+08	0,03223249	1,01E+08	0,03081705	2,75E+08	0,08440644						
		0,05568843	2,99E+09	0,92488394	7,03E+07	0,02173378	4,79E+07	0,01481534	1,25E+08	0,03856695						
		0,1226265	2,46E+09	0,84454323	9,68E+07	0,03319002	9,36E+07	0,03211076	2,63E+08	0,09015599						
	30°C	0,12724641	2,60E+09	0,83545449	1,17E+08	0,03777496	1,13E+08	0,03634737	2,81E+08	0,09042318						
		0,16608316	2,94E+09	0,80567052	1,01E+08	0,02773326	1,07E+08	0,02927244	5,02E+08	0,13732378						
		0,06374472	2,85E+09	0,90954176	8,55E+07	0,02731231	7,98E+07	0,02551593	1,18E+08	0,03762999						
		0,17775037	2,98E+09	0,79551556	1,06E+08	0,02838488	8,77E+07	0,02343244	5,71E+08	0,15266711						
		0,11847652	2,60E+09	0,85003891	9,86E+07	0,03227226	9,14E+07	0,02990919	2,68E+08	0,08777964						
pyruvate	4°C	0,12630914	6,51E+06	0,83883034	3,32E+05	0,04284903	1,47E+05	0,01888348	7,72E+05	0,09943714						
		0,12224189	5,80E+06	0,85968476	1,53E+05	0,02268019	5,98E+04	0,00885967	7,34E+05	0,10877538						
		0,16603191	6,08E+06	0,78954716	4,01E+05	0,05212245	2,23E+05	0,02901788	9,95E+05	0,12931251						
		0,12543316	6,20E+06	0,8384314	3,28E+05	0,04435177	1,46E+05	0,01970279	7,21E+05	0,09751404						
		0,19196017	9,69E+06	0,77691742	4,62E+05	0,03699689	2,42E+05	0,01937345	2,08E+06	0,16671224						
	21°C	0,11091093	6,90E+06	0,86128569	2,63E+05	0,03278171	1,43E+05	0,0178467	7,06E+05	0,0880859						
		0,12544698	7,85E+06	0,83496985	4,69E+05	0,04990505	1,78E+05	0,0189394	9,04E+05	0,09618569						
		0,1654237	9,15E+06	0,79757016	4,78E+05	0,04166119	3,18E+05	0,02769602	1,53E+06	0,13307262						
		0,07716135	1,04E+07	0,89975221	3,38E+05	0,02925167	1,24E+05	0,01075598	6,97E+05	0,06024014						
		0,17770562	6,23E+06	0,78973431	3,13E+05	0,03970073	1,44E+05	0,01827877	1,20E+06	0,1522862						
	30°C	0,15229364	7,43E+06	0,80769184	4,49E+05	0,0488763	2,05E+05	0,02229097	1,11E+06	0,1211409						
		0,17985211	9,52E+06	0,79032349	3,78E+05	0,03140433	3,21E+05	0,02666453	1,83E+06	0,15160765						
		0,06539577	9,49E+06	0,89789865	4,86E+05	0,04595095	1,92E+05	0,01821483	4,01E+05	0,03793557						
		0,24529049	8,49E+06	0,73037786	3,28E+05	0,02820846	1,93E+05	0,01657803	2,61E+06	0,22483565						
		0,13677009	5,70E+06	0,83338588	2,44E+05	0,03570308	1,24E+05	0,01812594	7,71E+05	0,1127851						
Glucose	4°C	0,07326483	6,52E+07	0,80348693	4,86E+06	0,05990746	4,65E+06	0,05731169	4,98E+06	0,06134479	8,40E+05	0,01035204	4,84E+05	0,00596693	1,32E+05	0,00163016
		0,0500451	6,15E+07	0,86278299	2,81E+06	0,03947758	3,24E+06	0,04541751	3,09E+06	0,04340732	3,59E+05	0,00504203	2,61E+05	0,00366745	1,46E+04	0,00020512
		0,09823198	5,70E+07	0,75032201	4,93E+06	0,0649774	5,81E+06	0,07648807	6,00E+06	0,07903618	1,16E+06	0,0152218	7,81E+05	0,01028466	2,79E+05	0,00366988
		0,06365309	7,73E+07	0,8320249	4,29E+06	0,0461564	4,75E+06	0,05118331	5,22E+06	0,05625025	7,84E+05	0,00844075	4,44E+05	0,00478455	1,08E+05	0,00115984
		0,09941368	6,43E+07	0,75167113	4,92E+06	0,05751378	6,76E+06	0,07903495	7,13E+06	0,08341891	1,27E+06	0,01482829	8,44E+05	0,00986915	3,13E+05	0,00366379
	21°C	0,03759658	6,71E+07	0,88844176	3,28E+06	0,04340841	2,11E+06	0,0279652	2,71E+06	0,03583987	2,34E+05	0,00310235	8,63E+04	0,00114279	7,53E+03	9,96E-05
		0,04681596	7,66E+07	0,86126252	4,89E+06	0,05493802	3,46E+06	0,03885476	3,12E+06	0,03503056	5,91E+05	0,00664378	2,70E+05	0,00304075	2,04E+04	0,00022961
		0,04631235	7,99E+07	0,87535784	3,13E+06	0,03433338	3,98E+06	0,0436263	3,24E+06	0,03548787	6,04E+05	0,00661405	3,76E+05	0,00411501	4,25E+04	0,00046554
		0,02264522	8,71E+07	0,92487043	3,52E+06	0,03737189	1,41E+06	0,01501953	2,12E+06	0,02249223	2,32E+04	0,00024592	0,00E+00	0	0,00E+00	0
		0,07199708	6,61E+07	0,81384606	4,11E+06	0,05055856	4,42E+06	0,05435381	5,19E+06	0,06385656	6,57E+05	0,00807957	5,69E+05	0,00700435	1,87E+05	0,0023011
	30°C	0,06139651	7,12E+07	0,82788554	4,91E+06	0,05707992	4,42E+06	0,05137429	4,44E+06	0,05165634	6,34E+05	0,00736903	3,18E+05	0,0037038	8,01E+04	0,00093107
		0,0745604	6,96E+07	0,79463457	6,02E+06	0,06865873	5,08E+06	0,05792467	5,62E+06	0,06408168	4,45E+05	0,00507881	6,52E+05	0,00743517	1,92E+05	0,00218636
		0,04272561	9,56E+07	0,87369014	5,31E+06	0,0485713	3,59E+06	0,03280365	4,34E+06	0,03969762	3,66E+05	0,00334431	1,81E+05	0,00165294	2,63E+04	0,00024005
		0,04881828	8,09E+07	0,86664186	3,72E+06	0,03986157	3,57E+06	0,03819921	4,47E+06	0,04785174	4,10E+05	0,004393	2,61E+05	0,00279328	2,42E+04	0,00025935
		0,05127394	6,48E+07	0,85388752	3,90E+06	0,05146572	3,02E+06	0,03979529	3,53E+06	0,04651053	3,89E+05	0,00512802	2,07E+05	0,00273386	3,63E+04	0,00047905
CisAco	4°C	0,07309987	1,61E+08	0,7547668	2,35E+07	0,10998297	2,02E+07	0,09471811	5,61E+06	0,02623361	2,35E+06	0,01101331	7,02E+05	0,0032852	0,00E+00	0
		0,03809628	1,54E+08	0,86430216	1,27E+07	0,07106839	6,83E+06	0,03822603	4,39E+06	0,02455648	3,30E+05	0,00184694	0,00E+00	0	0,00E+00	0
		0,07805008	1,04E+08	0,73360541	1,80E+07	0,12725908	1,19E+07	0,08396205	6,76E+06	0,04768903	1,04E+06	0,00737192	1,59E+04	0,00011251	0,00E+00	0
		0,09384896	1,30E+08	0,70427945	2,18E+07	0,11872662	1,95E+07	0,10617147	1,01E+07	0,05496623	2,24E+06	0,0121556	6,81E+05	0,00370062	0,00E+00	0
		0,08293087	1,66E+08	0,73278723	2,62E+07	0,11590234	1,90E+07	0,08406499	1,29E+07	0,05682201	2,04E+06	0,00903034	3,15E+05	0,0013931	0,00E+00	0
	21°C	0,11202823	1,15E+08	0,66543125	2,05E+07	0,11800902	2,28E+07	0,1315876	9,77E+06	0,05634623	3,67E+06	0,02118302	1,29E+06	0,00744287	0,00E+00	0
		0,11019469	6,34E+07	0,67179279	1,08E+07	0,1142768	1,21E+07	0,12846169	5,57E+06	0,05903608	1,82E+06	0,01930355	6,73E+05	0,0071291	0,00E+00	0
		0,08802143	1,23E+08	0,71561319	2,05E+07	0,11950615	1,79E+07	0,10467857	7,61E+06	0,04442006	2,21E+06	0,01290503	4,93E+05	0,002877	0,00E+00	0
		0,07808904	1,27E+08	0,73721684	1,99E+07	0,11577835	1,77E+07	0,10275372	5,56E+06	0,0322956	1,62E+06	0,00941577	4,37E+05	0,00253972	0,00E+00	0
		0,08381812	1,33E+08	0,72763309	2,12E+07	0,11601377	1,76E+07	0,09632345	8,75E+06	0,04789051	1,85E+06	0,01011933	3,69E+05	0,00201984	0,00E+00	0
	30°C	0,11399841	1,55E+08	0,66026469	2,88E+07	0,12270744	2,84E+07	0,1208179	1,67E+07	0,07137198	4,44E+06	0,01891487	1,33E+06	0,00566698	6,01E+04	0,00025615
		0,08564974	2,00E+08	0,73469363	2,93E+07	0,1076868	2,07E+07	0,07601943	1,97E+07	0,07222777	2,55E+06	0,00937237	0,00E+00	0	0,00E+00	0

		0,06196927	1,51E+08	0,78621059	1,95E+07	0,10190101	1,38E+07	0,07187516	6,59E+06	0,03441926	9,70E+05	0,00506338	1,02E+05	0,00053059	0,00E+00	0
		0,10804466	1,40E+08	0,68331263	2,17E+07	0,10625408	2,50E+07	0,12240581	1,27E+07	0,06234975	3,73E+06	0,01823562	1,52E+06	0,0074421	0,00E+00	0
		0,10127286	1,29E+08	0,68324717	2,36E+07	0,12516411	2,14E+07	0,11384772	1,12E+07	0,05968915	2,74E+06	0,01454909	6,60E+05	0,00350276	0,00E+00	0
		0,06548372	7,94E+07	0,81400409	7,58E+06	0,07773976	7,90E+06	0,0809896	2,19E+06	0,02244071	3,66E+05	0,00375168	1,05E+05	0,00107416		
		0,03642166	1,08E+08	0,89035591	6,03E+06	0,049516	5,86E+06	0,04814367	1,42E+06	0,01166475	3,50E+04	0,00028766	3,90E+03	3,20E-05		
	4°C	0,07390697	1,07E+08	0,79270859	1,12E+07	0,08310373	1,23E+07	0,09112304	3,94E+06	0,02911436	3,93E+05	0,00290942	1,41E+05	0,00104087		
		0,05862518	5,20E+07	0,83223047	4,35E+06	0,06973332	4,70E+06	0,07524014	1,17E+06	0,01869606	2,30E+05	0,00367594	2,65E+04	0,00042407		
		0,08456726	1,73E+08	0,76386256	2,15E+07	0,09487478	2,31E+07	0,10230381	7,64E+06	0,03379994	8,69E+05	0,00384042	2,98E+05	0,00131848		
		0,08623903	1,27E+08	0,77306867	1,34E+07	0,08132937	1,68E+07	0,1021005	5,18E+06	0,03140201	1,49E+06	0,0090385	5,05E+05	0,00306095		
		0,08771834	6,08E+07	0,76423293	6,98E+06	0,08770465	8,50E+06	0,10684171	2,42E+06	0,03043818	6,39E+05	0,00802359	2,20E+05	0,00275894		
Akg	21°C	0,08397925	1,19E+08	0,76290877	1,55E+07	0,09980129	1,58E+07	0,10138436	4,37E+06	0,02812826	9,24E+05	0,00594515	2,85E+05	0,00183217		
		0,07112723	9,71E+07	0,79010509	1,19E+07	0,09669293	1,07E+07	0,0872733	2,51E+06	0,02044765	5,35E+05	0,0043515	1,39E+05	0,00112953		
		0,0768623	1,17E+08	0,78206233	1,38E+07	0,09192999	1,39E+07	0,0925491	4,19E+06	0,02792275	6,25E+05	0,00416406	2,06E+05	0,00137176		
		0,10987054	1,43E+08	0,72180027	1,75E+07	0,08851332	2,55E+07	0,12851331	9,06E+06	0,04568507	2,12E+06	0,01068256	9,53E+05	0,00480547		
		0,08148232	2,41E+08	0,78014589	2,50E+07	0,08095507	2,95E+07	0,09550781	1,21E+07	0,03923806	9,40E+05	0,00303907	3,45E+05	0,00111409		
	30°C	0,06529121	1,72E+08	0,81158092	1,72E+07	0,08135849	1,70E+07	0,08037369	4,92E+06	0,02322295	5,58E+05	0,00263845	1,75E+05	0,0008255		
		0,10443119	1,43E+08	0,73873577	1,52E+07	0,07873372	2,42E+07	0,1252123	8,01E+06	0,04138362	2,10E+06	0,01082617	9,89E+05	0,00510841		
		0,09824363	1,52E+08	0,73891249	1,92E+07	0,09316029	2,44E+07	0,11820652	8,21E+06	0,0398324	1,50E+06	0,00729388	5,35E+05	0,00259442		
		0,06341597	286888837	0,82876647	21682925,9	0,06263779	27272306,6	0,07878443	8292534,64	0,02395553	1478928,82	0,00427234	548131,381	0,00158345		
		0,03367838	331452988	0,90480508	13438968,9	0,03668589	16124670,1	0,04401735	5236776,15	0,01429543	71891,4707	0,00019625	0	0		
	4°C	0,06427141	260044388	0,82366339	21424523,7	0,06785994	24194779,4	0,07663443	8878125,92	0,02812053	865660,009	0,00274189	309346,53	0,00097982		
		0,06087678	294779986	0,8382372	19936557,5	0,05669165	26413494,5	0,07510949	8353477,64	0,02375397	1697509,51	0,00482704	485525,404	0,00138064		
		0,06356442	260666302	0,83000383	18979433,6	0,0604336	24248133,1	0,07720999	8663884,72	0,02758722	1136866,58	0,00361997	359716,805	0,0011454		
		0,07468368	344642267	0,80950845	26150843,7	0,06142406	38484918	0,09039479	11460140,2	0,026918	3543344,86	0,00832274	1461129,66	0,00343196		
		0,08682854	376074978	0,77771972	35377192,2	0,07315972	50343782,2	0,1041105	15110781	0,03124896	4734267,11	0,00979042	1920067,05	0,00397068		
Gln	21°C	0,07287524	382367205	0,80663624	32006508,2	0,06752046	42900053,8	0,09050132	12987079,6	0,02739735	2873590,6	0,00606208	892372,749	0,00188254		
		0,07081984	399501605	0,81052632	33656010,6	0,06828279	43936757,7	0,08914082	11294514,6	0,02291481	3394041,88	0,00688598	1108656,1	0,00224929		
		0,06728367	392911171	0,8203832	29666434,8	0,06194236	41093186,1	0,08580097	12496740,4	0,02609271	2063211,83	0,00430791	705407,503	0,00147286		
		0,09212658	302294535	0,77932801	22970387,2	0,05921862	40677291,9	0,10486777	15469041,1	0,03987984	4456721,05	0,01148961	2023299,81	0,00521615		
		0,07305489	393107394	0,81885223	25563384,2	0,05324915	40452874,1	0,08426432	16441984	0,03424905	2965270,99	0,00617673	1540316,89	0,00320852		
	30°C	0,05619128	372832625	0,8478632	24354355	0,05538453	31193190,1	0,07093681	9160996,17	0,02083313	1632623,67	0,00371277	558262,243	0,00126955		
		0,07657544	281596210	0,81345958	17700621	0,05113258	32075468,8	0,09265784	9960273,34	0,02877269	3384053,05	0,00977567	1454488,68	0,00420165		
		0,0700353	296673174	0,8223447	19281946	0,05344739	31208990,3	0,08650781	10672563,6	0,02958315	2027744,6	0,00562068	900566,49	0,00249627		
		0,06791057	7,96E+08	0,80461735	8,42E+07	0,08504649	8,19E+07	0,08272888	2,22E+07	0,02244637	4,05E+06	0,0040951	1,05E+06	0,00106582		
		0,03329294	8,57E+08	0,89909588	4,53E+07	0,04751177	3,98E+07	0,04174601	1,06E+07	0,01112443	4,97E+05	0,0005219	0,00E+00	0		
	4°C	0,07070304	8,91E+08	0,79362876	1,04E+08	0,09282937	9,50E+07	0,08457789	2,81E+07	0,02503278	3,62E+06	0,00322424	7,94E+05	0,00070695		
		0,08384185	7,76E+08	0,76590374	9,92E+07	0,0979273	9,89E+07	0,09762714	3,04E+07	0,02999266	6,79E+06	0,00669613	1,88E+06	0,00185304		
		0,06585412	8,47E+08	0,81063	8,60E+07	0,08232955	8,24E+07	0,07890299	2,53E+07	0,02423403	3,22E+06	0,00308421	8,56E+05	0,00081923		
		0,09360665	7,85E+08	0,75175496	9,65E+07	0,09233367	1,13E+08	0,10778243	3,72E+07	0,03558397	9,76E+06	0,00934208	3,35E+06	0,0032029		
		0,09452676	8,05E+08	0,74315315	1,10E+08	0,10144947	1,20E+08	0,11043938	3,50E+07	0,03235549	1,06E+07	0,00977345	3,06E+06	0,00282906		
Glu	21°C	0,0866269	9,77E+08	0,75607241	1,32E+08	0,10195999	1,36E+08	0,10519019	3,62E+07	0,02799726	9,17E+06	0,00709842	2,17E+06	0,00168174		
		0,06739671	8,01E+08	0,80574993	8,16E+07	0,08202624	8,80E+07	0,08848411	1,80E+07	0,01810246	4,48E+06	0,0045046	1,13E+06	0,00113266		
		0,07147913	8,51E+08	0,79550819	9,35E+07	0,08741482	9,38E+07	0,0876975	2,57E+07	0,02402895	4,55E+06	0,00425375	1,17E+06	0,00109679		
		0,08693024	6,36E+08	0,76628566	7,30E+07	0,08803682	8,57E+07	0,10334861	2,64E+07	0,0318716	6,62E+06	0,00798419	2,05E+06	0,00247312		
		0,05856525	8,50E+08	0,83753651	6,46E+07	0,0636039	7,15E+07	0,07041524	2,63E+07	0,02590006	2,06E+06	0,00202978	5,22E+05	0,00051451		
	30°C	0,05410128	8,78E+08	0,84011556	7,50E+07	0,07181005	7,19E+07	0,06880363	1,73E+07	0,0165068	2,35E+06	0,00225113	5,36E+05	0,00051283		
		0,08318113	6,67E+08	0,77995126	6,65E+07	0,07780577	8,69E+07	0,10159784	2,61E+07	0,03047979	6,30E+06	0,00736186	2,40E+06	0,00280348		
		0,07885298	6,09E+08	0,78534048	6,41E+07	0,08261601	7,25E+07	0,09345224	2,44E+07	0,03143433	4,15E+06	0,0053433	1,41E+06	0,00181364		
		0,10171765	4,16E+08	0,75688859	6,76E+07	0,12278509	4,58E+07	0,08332315	1,68E+07	0,03057349	3,54E+06	0,00642969				
		0,0488418	5,44E+08	0,87381898	4,35E+07	0,06996789	2,75E+07	0,04424011	6,83E+06	0,01097297	6,22E+05	0,00100004				
	4°C	0,10796812	4,31E+08	0,73648295	7,72E+07	0,13187168	5,80E+07	0,09907652	1,66E+07	0,0284276	2,42E+06	0,00414124				
		0,11989436	3,99E+08	0,70717512	8,53E+07	0,15095139	5,85E+07	0,10358047	1,79E+07	0,03170698	3,72E+06	0,00658604				
		0,10216784	4,97E+08	0,74908796	8,59E+07	0,12951798	5,95E+07	0,08970698	1,79E+07	0,0270089	3,10E+06	0,00467818				
		0,13995986	3,84E+08	0,67621459	8,54E+07	0,15056287	6,81E+07	0,12008521	2,47E+07	0,04344318	5,50E+06	0,00969415				
		0,13611049	3,88E+08	0,67557243	9,21E+07	0,1602626	6,80E+07	0,11820419	2,07E+07	0,03607213	5,69E+06	0,00988865				
Succ	21°C	0,11466496	4,35E+08	0,71828272	8,67E+07	0,14326817	6,39E+07	0,10567678	1,64E+07	0,02705122	3,46E+06	0,00572111				
		0,09038664	4,37E+08	0,76942388	7,23E+07	0,12734181	4,51E+07	0,0794083	1,13E+07	0,01991591	2,22E+06	0,0039101				
		0,0950458	4,63E+08	0,75676919	8,32E+07	0,13599504	4,99E+07	0,0816408	1,31E+07	0,0214733	2,52E+06	0,00412167				
		0,1326971	4,31E+08	0,69121333	9,05E+07	0,14499317	7,12E+07	0,11403688	2,58E+07	0,04130502	5,27E+06	0,0084516				
		0,11798587	4,27E+08	0,74645118	5,87E+07	0,1026292	5,19E+07	0,09070231	3,03E+07	0,05295956	4,15E+06	0,00725774				
	30°C	0,07827972	4,26E+08	0,80050333	5,78E+07	0,10865101	3,73E+07	0,07010655	9,95E+06	0,01870166	1,08E+06	0,00203745				
		0,13412994	3,78E+08	0,69549897	7,88E+07	0,14514848	5,31E+07	0,09784458	2,73E+07	0,05034976	6,06E+06	0,01115821				
		0,12589138	4,30E+08	0,70784933	8,16E+07	0,13438354	6,82E+07	0,11241557	2,25E+07	0,03705538	5,04E+06	0,00829617				

		0,09028163	2,93E+08	0,785793	4,10E+07	0,10981984	2,48E+07	0,06636517	1,25E+07	0,03351163	1,68E+06	0,00451036
		0,03481004	2,82E+08	0,90830856	1,63E+07	0,0525568	9,60E+06	0,03092109	2,49E+06	0,008013	6,23E+04	0,00020055
	4°C	0,08175299	2,66E+08	0,7905228	4,01E+07	0,11901038	2,21E+07	0,06572431	7,55E+06	0,02241707	7,83E+05	0,00232543
		0,10446006	2,79E+08	0,75186664	4,65E+07	0,12538883	3,01E+07	0,08125818	1,33E+07	0,03601031	2,03E+06	0,00547603
		0,07927805	2,23E+08	0,81113496	2,64E+07	0,09603861	1,67E+07	0,06087516	7,83E+06	0,02848179	9,54E+05	0,00346947
		0,12255489	3,68E+08	0,71879568	6,69E+07	0,13087142	5,12E+07	0,10021462	2,12E+07	0,04155423	4,38E+06	0,00856406
		0,13208973	3,34E+08	0,68284829	8,13E+07	0,16602638	4,85E+07	0,09912819	2,15E+07	0,04391242	3,96E+06	0,00808473
Asp	21°C	0,10962785	3,16E+08	0,72479898	6,64E+07	0,15218308	3,81E+07	0,08734069	1,36E+07	0,03106208	2,01E+06	0,00461518
		0,07958766	3,27E+08	0,79865404	4,58E+07	0,11180567	2,66E+07	0,06503349	8,83E+06	0,0215492	1,21E+06	0,0029576
		0,08478534	3,02E+08	0,79208347	4,15E+07	0,10910962	2,66E+07	0,06989365	9,67E+06	0,02540861	1,33E+06	0,00350465
		0,11004197	2,83E+08	0,74545683	4,65E+07	0,12245059	3,27E+07	0,08602027	1,47E+07	0,03861246	2,83E+06	0,00745985
		0,07658938	2,62E+08	0,82891195	2,50E+07	0,0791985	1,66E+07	0,05250507	1,12E+07	0,03538902	1,26E+06	0,00399545
	30°C	0,06648654	3,17E+08	0,83110807	3,58E+07	0,0938499	2,09E+07	0,05469994	7,12E+06	0,018672	6,37E+05	0,00167009
		0,12656863	2,75E+08	0,7223674	4,59E+07	0,1208174	3,63E+07	0,09551672	1,93E+07	0,05077023	4,00E+06	0,01052825
		0,09189066	2,33E+08	0,78483729	3,07E+07	0,10319753	2,27E+07	0,07647094	9,08E+06	0,03055374	1,47E+06	0,0049405
